# High atmospheric temperatures and ‘ambient incubation’ drive embryonic development and lead to earlier hatching in a passerine bird

**DOI:** 10.1098/rsos.150371

**Published:** 2016-02-03

**Authors:** Simon C. Griffith, Mark C. Mainwaring, Enrico Sorato, Christa Beckmann

**Affiliations:** 1 Department of Biological Sciences, Macquarie University, Sydney, New South Wales 2109, Australia; 2 School of Biological and Earth Sciences, University of New South Wales, Sydney, New South Wales 2052, Australia; 3 Centre for Integrative Ecology, School of Life and Environmental Sciences, Deakin University, Geelong, Victoria 3216, Australia

**Keywords:** nest temperature, ambient incubation, nest architecture, nest microclimate, *Taeniopygia guttata*, zebra finch

## Abstract

Tropical and subtropical species typically experience relatively high atmospheric temperatures during reproduction, and are subject to climate-related challenges that are largely unexplored, relative to more extensive work conducted in temperate regions. We studied the effects of high atmospheric and nest temperatures during reproduction in the zebra finch. We characterized the temperature within nests in a subtropical population of this species in relation to atmospheric temperature. Temperatures within nests frequently exceeded the level at which embryo’s develop optimally, even in the absence of parental incubation. We experimentally manipulated internal nest temperature to demonstrate that an average difference of 6°C in the nest temperature during the laying period reduced hatching time by an average of 3% of the total incubation time, owing to ‘ambient incubation’. Given the avian constraint of laying a single egg per day, the first eggs of a clutch are subject to prolonged effects of nest temperature relative to later laid eggs, potentially increasing hatching asynchrony. While birds may ameliorate the negative effects of ambient incubation on embryonic development by varying the location and design of their nests, high atmospheric temperatures are likely to constitute an important selective force on avian reproductive behaviour and physiology in subtropical and tropical regions, particularly in the light of predicted climate change that in many areas is leading to a higher frequency of hot days during the periods when birds breed.

## 1. Introduction

Long-term monitoring of reproduction in populations of several well-studied insectivorous passerine species in temperate regions of Europe and North America has provided some of the best empirical evidence of animal adaptation to climatic variation [1]. Reproductive success in such species is typically reliant on the coordination of breeding with a short time period of high insect abundance, and many bird species have shifted the timing of egg-laying in line with the changing phenology of their prey [2–4]. However, while these long-term studies of seasonally breeding bird species in the temperate regions of the Northern Hemisphere have provided some classic case studies of selection and adaptation to climatic variation, the geographical and taxonomic biases associated with these studies may limit our ability to predict the broader routes through which climate affects avian reproduction in other regions of the world [5–7].

**Figure 1.**
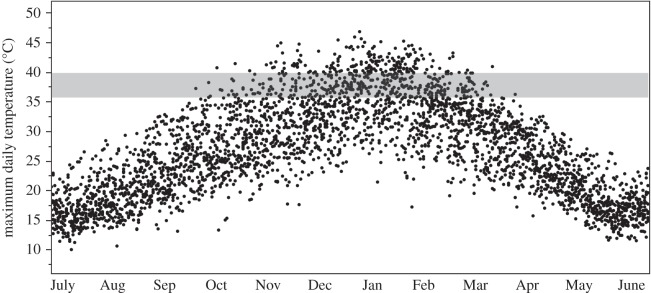
The maximum daily atmospheric temperature (°C) at the study site recorded on each day of the year between 2005 and 2013 (data from the Bureau of Meteorology). Each point represents an individual daily maximum temperature in that period. The shaded section highlights the temperature range that is ideal for the development of avian embryos.

There are concerns that tropical birds may be negatively affected by climatic variation in ways currently unforeseen as a consequence of our reliance on studies of relatively few intensively studied species in temperate environments [7]. An obvious example is that the direct effect of nest temperature on development and reproductive success is unlikely to be a major selective force for temperate species that typically breed within a three to four month time window, when atmospheric temperatures typically range between an average of 5–20°C [8,9]. By contrast, birds breeding in tropical and subtropical regions typically breed across seven to eight months of the year, when atmospheric temperatures may range between −5 and 45°C [10] (figure 1). Egg viability has been shown to be adversely affected when eggs are left for a number of days in the pre-incubation stage at high ambient temperatures, owing to direct effects of temperature on the albumen and also by providing better conditions for harmful microorganisms [11,12]. A couple of recent studies in Australia [13,14] and one in South Africa [15] have also demonstrated the adverse effect of high temperatures on the growth rate of nestling passerines either owing to direct effects on the nestlings themselves or through the inhibited foraging activities of their parents.

Here, we consider the effect of hot nest conditions on embryonic development. In passerine birds, the optimal temperature for embryo development is 36–40°C and prolonged exposure to temperatures above 40.5°C is apparently lethal to developing embryos [16,17]. As such, there may be a real challenge for tropical and subtropical species (where atmospheric temperatures often exceed 36–40°C) in reducing the negative effects of high temperatures on developing embryos. Even when nest temperatures are below the lethal threshold, ‘ambient incubation’—where eggs left unattended during the laying period prior to incubation, begin to develop—might have negative fitness effects by impairing the ability of parents to optimize the development rate and the degree of competition within their broods [18,19]. This is likely to be a problem, because birds lay only one egg per day and the early laid eggs spend several days in the nest before the clutch is complete and parents start to attend their nest. To date, such ambient incubation has not been considered by studies performed in temperate regions [1,8], probably because atmospheric temperatures at higher latitudes are rarely high enough to support embryonic development. However, ambient incubation may affect many avian species in hotter parts of the world, and is something that should be considered as a potential part of the threatening process of climate change on birds [7], although there may also be benefits of breeding in warmer conditions [20,21].

Here, we study the effect of high atmospheric temperature on a desert bird, the zebra finch (*Taeniopygia guttata*), that breeds in an environment in which the atmospheric temperature regularly exceeds 36°C, the lower threshold for optimum embryonic development (figure 1). Unless the temperature of the nest chamber is buffered against extreme atmospheric conditions, it seems possible that in this and other bird species living in similar climates, embryos may be considerably affected by atmospheric temperature in the period between egg-laying and the start of parental incubation.

We first characterize the natural variation in nest temperature, and modify nest architecture to investigate the effect of nest structure and positioning on the internal temperature of the nest in relation to atmospheric temperature. We then experimentally manipulated the temperature of nest chambers in which eggs were held between laying and the start of parental incubation to quantify the effect of nest temperature on the period required for eggs to hatch. By systematically assigning eggs from clutches to either ‘hot’ or ‘cool’ experimental treatments during the egg-laying period and then returning all eggs to their original nests, we test the hypothesis that high nest temperatures during the laying period reduces the hatching time of individual eggs.

## 2. Material and methods

### 2.1 Study site and species

The data were collected during three time periods across the breeding season in the Austral spring and summer; October 2010, January–February 2012 and November–December 2012; from a population of wild zebra finches breeding in nest-boxes at the Fowlers Gap Arid Zone Research Station in far-western New South Wales, Australia (31°05^′^ S, ‘42°42^′^ E; for further details of the study site and nest-boxes, see [22]). The research station is located in the semi-arid zone of southeastern Australia, and the local weather patterns reflect those experienced across the wider Australian arid biome [10]. Atmospheric temperature varies seasonally, increasing as early spring advances through to summer (figure 1), whereas rainfall is low (approx. 240 mm annually), and unpredictable, often occurring in a few heavy precipitation events without a distinct seasonal pattern [10,22].

The zebra finches at this site have an average clutch size of 4.87 (±1.05 s.d.) and in three out of four earlier years studied, they bred from August to mid-December [22]—the months during which most of the work reported here was conducted. In the wild zebra finch, parents only start to incubate their eggs on the day that the clutch is completed (i.e. on the fifth day after the first egg is laid for a five egg clutch), and typically incubate for about 12 days before the eggs hatch [23]. Both male and female share incubation quite equitably and both start to incubate the clutch at the same rate on the same day [23].

### 2.2 Atmospheric and environmental temperatures

Daily atmospheric temperature data (in the shade) were obtained from the Australian Bureau of Meteorology’s Automated Weather Station at Fowlers Gap, located within 20 km of the study sites for the period from October 2004 to March 2013. Location temperature was recorded at 74 locations in the habitat occupied by the zebra finches in the period 2–6 February 2012 by placing iButtons (Maxim: DS1922L) within acacia scrub in sites where zebra finches could have nested, and other sites nearby where they typically do not nest such as locations on the ground, in short grasses and in low bushes. These 74 locations were all within an area of approximately 1 ha, and the design of this component of work was to look at the variation within a small area of habitat depending on the characteristics of the site selected (rather than between areas). The iButtons were placed to capture the variation with respect to both their height and orientation within grasses, and bushes (less than 5 m off the ground). To ensure the accuracy of temperature recording, before use, we placed all of the iButtons in an incubator set to 32°C for 24 h (and set to record temperature once a minute). All iButtons were then downloaded, and we checked the data to ensure all were functioning with the range specified by the manufacturer. All iButtons used read within 0.5°C of each other and the temperature to which the incubator was set (32°C). Given this level of accuracy, we did not correct individual iButton data for this relatively small variation.

### 2.3 Nest temperature

We placed a single iButton in each of 24 recently vacated (i.e. unoccupied) natural nests and 64 recently vacated nests in nest-boxes with the temperature recorded hourly for the periods 13–20 October 2010 and 27 January–6 February 2012. Natural zebra finch nests are typically positioned in the outermost branches and stems of acacia bushes and are made from grass stems with the nest chamber covered by a domed roof, and the chamber lined with a sparse covering of flower heads and feathers [24]. At this study site zebra finches readily adopt nest-boxes, in which they also construct a similar-sized nest complete with a grass roof on the inside of the nest-box [22]. All of the work on natural nests and nest-boxes was conducted in the Gap Hills area at Fowlers Gap, and in an area of suitable habitat that is approximately circular and has a diameter of about 2.6 km [25]. These nests varied in their condition, but most were soiled with excrement produced by the previous brood, and this may affect the thermal dynamics of the nest, as would the fact that they contained no eggs.

We quantified the location and design of natural nests, and nests in nest-boxes to examine their role in determining nest temperature. We also considered the effect of positioning and orientation on internal nest temperatures. Nest height was quantified as the distance between the top of the domed nest and the ground (cm), and nest orientation was measured in degrees from magnetic north (0–360°), and later categorized into north, east, south and west (NESW). All natural zebra finch nests that we have observed in the wild over the past 10 years of studying this species have been domed structures with a roof constructed of grass built over the nest cup (as also described by Zann [26]). To understand the relevance and importance of this roof as an adaptive component to reduce the effect of atmospheric temperatures and solar radiation on nest contents, we experimentally removed the roof from a number of nests, thus deliberately going outside the natural variation of nest structure in this species. The effect of the roof on natural nests’ temperature was examined by measuring temperature (limited to the period 13–20 October 2010) in four nests that had their roofs removed and in four intact (control) nests all of which were left in the position in which they were built.

### 2.4 Experimental manipulation of pre-incubation nest temperature

In the Austral spring of 2012 (September–November), all nest-boxes within the study site were monitored for reproductive activity as part of ongoing research. In the same area as zebra finches were nesting, we created four experimental nest chambers to manipulate the thermal environment that freshly laid eggs were subject to. The ‘hot’ nests consisted of a natural nest and a nest-box nest placed on stakes in a location that was exposed to the sun throughout the day, whereas the ‘cool’ chambers consisted of a natural nest and a nest-box nest placed on stakes in a fully shaded location under a tree, with virtually no direct solar radiation throughout the day. The positioning of these nests was done in a way to represent the two ends of the natural variation in positioning in cool and hot locations. The nests used had not been used for the rearing of chicks (during which they become very soiled), and therefore, structurally, they were the same as clean nests in which eggs are laid. The two nest-boxes had their entrances covered with wire mesh, and the natural nests were completely encased in balls of wire mesh to prevent zebra finches, or any other animals, from entering them while enabling both airflow and solar radiation to act on the nest.

A total of 33 eggs, from eight clutches, were removed on the day on which they were laid, marked with an individually recognizable number and replaced with a dummy egg made from white FIMO polymer modelling clay (Eberhard Faber, Neumarkt, Germany). All removed eggs were placed into one of the four experimental nest chambers. Using a paired design, every other egg from within a clutch was placed into either a ‘hot’ chamber or a ‘cool’ chamber, with alternate nests having their first laid eggs placed into the ‘hot’ or ‘cool’ chambers as appropriate in order to achieve a balanced design. We recorded the temperature every 10 min in the experimental nest chambers, using iButtons placed adjacent to the eggs. The treatment was conducted within the period 26 November–4 December. The average daily maximum temperature during this period was 37.27°C (±6.1 s.d.). This period is representative of the maximum daily temperatures recorded during November and December over the longer term data provided by the Bureau of Meteorology for this area (shown in figure 1), and the average daily maximum temperature is equivalent to the 80% quantile. For each nest, the treatment began on the day the first egg in a clutch was laid, removed and placed into the experimental chamber. Eggs across the laying sequence were thus exposed to different amounts of time in the chamber (as under natural conditions; [23]), with the first egg being inside the chamber for a day longer than the second egg and so on. All eggs were returned to their original nests, the day after the final egg was laid, allowing parents to incubate the experimentally treated clutch until hatching. Nests were checked daily for hatching from 10 days of parental incubation onwards, and the hatch time and date of all eggs were recorded, in order to calculate development duration from the start of parental incubation. Hatching checks were conducted between 08.00 and 10.00.

### 2.5 Statistical analyses

The data were analysed in the statistical package R (version 2.11.1) [27]. We examined differences in temperature between nesting locations (natural nests and nest-boxes) and the environment using generalized least-squares models (GLS) implemented in the R package nlme (function ‘gls’, [28]), with ‘temperature’ as the dependent variable, ‘atmospheric temperature’ as a fixed covariate, ‘location’ (natural nests, nest-boxes or environment) and ‘time of day’ (four levels: 0.00–06.00, 06.00–12.00, 12.00–18.00, 18.00–00.00) as fixed factors. Correlations between multiple observations from the same site and from the same day were modelled with a compound symmetric correlation structure, and heteroscedasticity of location temperature was modelled by specifying a linear increase of its variance with atmospheric temperature and by allowing different variances per type of location.

We examined variation in temperature between natural nests in relation to nest location and design, first, by using GLS models with ‘nest temperature’ as the dependent variable, ‘atmospheric temperature’ as a fixed covariate, ‘height’, ‘orientation’ (categorized as: ‘north’, ‘east’, ‘south’, ‘west’) and ‘time of day’ as fixed factors. As in the previous analyses, correlations between multiple observations from the same site and from the same day were modelled with a compound symmetric correlation structure, and heteroscedasticity of nest temperature was modelled by specifying a linear increase of variance with atmospheric temperature.

To assess the effect of roof removal on nest temperature, we used GLS models which had ‘nest temperature’ as the dependent variable, ‘atmospheric temperature’ as a fixed covariate, ‘nest type’ (control, experimental) and ‘time of day’ as fixed factors; heteroscedasticity of nest temperature and correlations between observations from the same nests and days were modelled as above.

We examined how nest microclimate influenced the hatching time of individual eggs by using linear-mixed models (with normal error distribution and identity link function), to assess differences in temperature between ‘holding chambers’ in each treatment (‘hot’ and ‘cool’) and between ‘holding chambers’ of different type (‘nest-boxes’ and ‘natural nests’); ‘chamber temperature’ featured as the dependent variable, ‘treatment’ and ‘chamber type’ constituted fixed effects, and ‘day’ represented a random effect. For these analyses, we did not control for variation in atmospheric temperature given the temporally balanced design. We then examined the length of time that eggs in different experimental chambers remained at ‘normal’ (36–40°C) and ‘lethal’ (more than 40.5°C) incubation temperatures, respectively, by running two sets of models, the first examining the number of hours per day during which chamber temperature was within the ‘normal’ range, the second modelling the number of hours per day during which chamber temperature was within the ‘lethal’ temperature range. In both sets of models, ‘treatment’ and ‘chamber type’ featured as fixed effects. To accommodate the high frequency of zero observations (zero-inflation, owing to the high number of days during which the chambers never reached ‘normal’ or ‘lethal’ incubation temperatures, respectively), we used hurdle models, implemented in the R package pscl [29]. Presence (more than 0 h)–absence (0 h) was first modelled with a Bernoulli distribution, whereas a zero-truncated Poisson or negative-binomial distribution models accounted for the abundance of presence data [30]. The final model was obtained by multiplying the predictions from both steps. Akaike information criterion (adapted for small sample sizes, AICc; see below) was used to select the best distribution (zero-truncated Poisson or zero-truncated negative-binomial) for abundance models.

Lastly, we examined variation in the hatching time of individual eggs using linear-mixed models with ‘hatching time’ as the dependent variable, experimental ‘treatment’ and ‘chamber type’ as fixed factors, ‘egg number’ (1–5, indicating position in the laying sequence) as a fixed covariate, and ‘identity of nest of origin’ as a random effect.

To facilitate interpretability and comparison of effect sizes, all model predictors were centred by subtraction of mean values: before centring, factors were binarized by splitting them into dummy variables (0,1) and then centred on their mean values; and continuous variables were further standardized by dividing them by twice their sample standard deviation. For models with ‘atmospheric temperature’ as a covariate, nonlinear effects were modelled by the inclusion of quadratic and cubic terms. Sets of candidate models were defined to reflect multiple working hypotheses [31], guided by knowledge of the study system and hypothesized predictors. More complex models (e.g. including two-way and three-way interactions) were excluded if they had higher AICc scores than simpler nested models. Inference was then based on the models with the lowest AICc scores (‘best models’), in all cases the remaining models had *Δ*AICc≥5 and accordingly had considerably less support than best models (see tables S1–S3 in the electronic supplementary material). Fixed effect estimates for continuous variables and estimated marginal means for factors are presented ±1 standard error. All data from this study are archived at Dryad and accessible at doi:10.5061/dryad.7vf87. The R scripts used for the analysis are also available in the electronic supplementary material.

## 3. Results

### 3.1 Atmospheric temperature variation at the study site

Maximum daily temperature records from the study site over a period of eight and a half years revealed that atmospheric conditions regularly exceed the temperature at which eggs are normally incubated by their parents (36°C; figure 1). Daily maximal temperatures of more than 36°C occurred in each month between October and March of the following year (i.e. from mid-spring, through summer and into early-autumn). In October (the peak month of zebra finch breeding at the study site; [22]), this threshold was reached on 6% of days (17 days out of 286) for which temperature data were available, whereas it was reached on 58% of days during January (179 out of 307 days). Atmospheric temperatures also regularly exceeded the temperature of 40.5°C that is considered lethal to avian eggs. During the period analysed (October 2004–March 2013), the atmospheric daily maximum temperature exceeded 40.5°C on 141 days (out of a total of 3385 days; 4%), with the highest maximum temperature of 46.8°C being recorded on 5 January 2013. These days of extreme heat (more than 40.5°C) were distributed across the months as follows: October 1%; November 16%; December 13%; January 46%; February 21%; March 3%.

### 3.2 Variation in microclimate in the habitat around nesting sites

**Figure 2.**
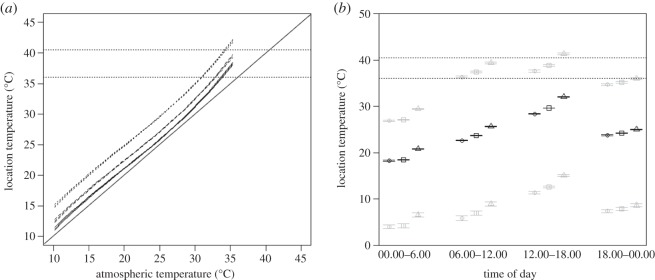
(*a*) The predicted temperature (±s.e.) at different locations (‘natural nests’, *n*=24, solid line; ‘nest-boxes’, *n*=64, dashed line; ‘vegetation’, *n*=74, dotted line) during the hottest part of the day (12.00–18.00) as a function of atmospheric temperature. The horizontal dotted lines indicate lethal (40.5°C) and normal (36°C) incubation temperatures, respectively, whereas the solid black line indicates the line 1 : 1, represents the hypothetical situation where atmospheric temperature and the nest temperature are equal. (*b*) Predicted nest temperatures for the three different locations (natural nests, nest-boxes, vegetation) during different parts of the day; ‘natural nests’, ‘nest-boxes’ and ‘vegetation’ are represented by ‘circles’, ‘squares’ and ‘triangles’, respectively. Location temperatures are predicted for mean (‘black’ medial series), maximum (‘grey’ top series) and minimum (‘grey’ bottom series) atmospheric temperature values recorded during each time period; vertical bars represent 1 s.e. Top and bottom dotted horizontal lines indicate lethal (40.5°C) and normal (36°C) incubation temperatures, respectively.

Irrespective of location type (natural nests, nests in nest-boxes or in habitat locations), location temperature increased in a slightly nonlinear fashion with atmospheric temperature (table 1 and figure 2*a*). However, while both actual nests (natural nests and nests in nest-boxes) and locations in habitat, were, on average hotter than the atmospheric temperature, natural nests were cooler than nest-boxes, which in turn had a lower temperature than sampled points in the local environment (table 1).

**Table 1.** Final best model for location temperature across location types and time of day, controlled for atmospheric temperature (‘atm.T’). (The superscripts ‘2’ and ‘3’ indicate quadratic and cubic effects, respectively. Colon symbolizes interactions; double asterisks indicate highly significant (*p*<0.01) predictors. All factors were binarized by splitting them into dummy variables (0,1 and centred by subtraction of their mean value; because *n* levels can be coded using *n*−1 dummy variables, ‘night’ and ‘vegetation’ effects are specified by negative values on the remaining dummy variables (corresponding to 0 s in the uncentred dummy variables). Continuous variables are standardized by subtracting their mean and by dividing them by twice their sample standard deviation.)

predictor	estimate	s.e.	*t*-value	*P*
final best model: T∼1+atm.T+atm.T^2^+atm.T^3^+time of day+location+time of day×location				
intercept	24.17	0.02	1105.15	<0.01**
atm.T	10.90	0.09	120.21	<0.01**
atm.T^2^	1.11	0.14	7.99	<0.01**
atm.T^3^	1.20	0.15	8.17	<0.01**
morning (06.00–12.00)	3.40	0.03	99.64	<0.01**
afternoon (12.00–18.00) evening	3.98	0.04	102.79	<0.01**
evening (18.00–24.00)	0.75	0.04	20.62	<0.01**
natural nest	−2.71	0.06	−43.10	<0.01**
nest-box	−1.98	0.05	−41.24	<0.01**
natural nest : morning	−0.57	0.11	−5.31	<0.01**
natural nest : afternoon	−1.20	0.11	−10.46	<0.01**
natural nest : evening	1.27	0.11	11.35	<0.01**
nest-box : morning	0.34	0.07	4.62	<0.01**
nest-box : afternoon	−0.09	0.09	−1.02	0.31
nest-box : evening	1.58	0.08	19.08	<0.01**

As expected, location temperatures also varied during the day, with an increase from morning to late afternoon followed by a decrease through the evening-night (table 1). However, average diel temperature values did not vary in the same fashion across all location types (table 1 and figure 2*b* medial series): during the coolest part of the day (00.00–06.00; mean atmospheric temperature=20.3°C, s.d.=4.5, range=7–29°C), natural nests and nest-boxes had similar temperatures (mean *T*=18.2°C and 18.4°C, respectively), and were both cooler than the sampled points in the vegetation (mean *T*=20.8°C). On the other hand, during the hottest period of the day (12.00–18.00; mean atmospheric temperature=27.2°C, s.d.=4.9, range=10.2–35°C), natural nests were significantly cooler than nest-boxes (mean *T*=28.3°C and 29.6°C, respectively), which in turn were cooler than sampled vegetation points in the local environment (mean *T*=32.1°C). At the maximum atmospheric temperature (35°C; 12.00–18.00) recorded in the course of this part of the study, the microclimate at different locations is predicted to be 37.5°C in natural nests, 38.2°C in nest-boxes and 41.5°C in the vegetation (figure 2*b* top series). More generally, at the maximum atmospheric temperatures recorded during each period of the day (00.00–06.00=29°C; 06.00–12.00=34.2°C; 12.00–18.00=35°C; 18.00–00.00=34.3°C), the predicted average temperature at all three location types exceeded the incubation threshold (36°C) during the daytime, though only the vegetation temperature exceeded the lethal threshold during the hottest part of the day (afternoon; figure 2*b*, top series).

### 3.3 Nest characteristics and their microclimates

**Figure 3.**
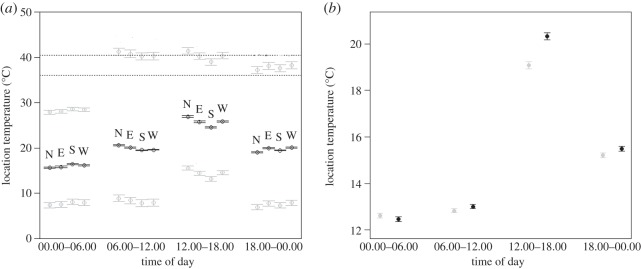
(*a*) Predicted temperatures for natural nests as a function of nest orientation (‘E’: *n*=4, ‘N’: *n*=3, ‘S’: *n*=6, ‘W’: *n*=6 nests) and the time period in the day. Nest temperatures are predicted for mean (‘black’ medial series), maximum (‘grey’ top series) and minimum (‘grey’ bottom series) atmospheric temperature values recorded during each time of day period; data were collected in October 2010 (11 days) and January–February 2012 (11 days). Vertical bars represent 1 s.e. Top and bottom dotted horizontal lines indicate lethal (40.5°C) and normal (36°C) incubation temperatures, respectively. (*b*) Predicted nest temperatures at different periods of the day for natural nests that are either experimental nests without roofs (*n*=4; ‘black’ symbols) or control nests with intact roofs (*n*=4; ‘grey’ symbols); data were collected in October 2010 (average atmospheric temperature=15.9°C). Vertical bars represent 1 s.e.

Height from the ground of the measured nests averaged 162 cm (±7.4 s.e.), with a range of 57–261 cm, but this did not influence the internal temperature of these nests (table 2*a*). However, nest temperature did vary with the orientation of the entrance hole and time of day (table 2*a* and figure 3*a*). The effect of the orientation of the entrance hole on nest temperature was strongest during the hottest period of the day (12.00–18.00; table 2*a*: east=−1.33±0.29, south=−3.11±0.25, west=−1.59±0.24); moreover, temperature was highest for southerly exposed/oriented nests and lowest for northerly oriented ones during the night (00.00–06.00; table 2*a*), but the opposite pattern was apparent during the daytime, particularly between 12.00 and 18.00.

**Table 2.** (*a*) Final best model for temperature of natural nests according to nest orientation, and time of day, controlled for atmospheric temperature (‘atm.T’); nest height was also considered as a predictor but did not feature in the best model. (*b*) Final best model for temperature of natural nests according to nest structure (‘roof removed’ versus ‘control’) and time of day, controlled for atmospheric temperature (‘atm.T’). (The superscripts ‘2’ and ‘3’ indicate quadratic and cubic effects respectively. Colon symbolizes interactions; double asterisks indicates highly significant (*p*<0.01) predictors. All factors were binarized by splitting them into dummy variables (0,1) and were centred by subtraction of their mean value; because *n* levels can be coded using *n*−1 dummy variables, ‘night’ and ‘control nests’ effects are specified by negative values on the remaining dummy variables (corresponding to 0 s in the uncentred dummy variables). Continuous variables are standardized by subtracting their mean and by dividing them by twice their sample standard deviation.)

predictor	estimate	s.e.	*t*-value	*p*
(*a*) final best model: T∼1+atm.T+atm.T^2^+atm.T^3^+time of day+orientation+orientation: time of day+ atm.T: time of day				
intercept	20.12	0.05	408.09	<0.01**
atm.T	12.13	0.27	44.64	<0.01**
atm.T^2^	2.30	0.41	5.58	<0.01**
atm.T^3^	1.04	0.39	2.63	<0.01**
morning (06.00–12.00)	2.71	0.08	34.44	<0.01**
afternoon (12.00–18.00) evening	3.51	0.10	34.67	<0.01**
evening (18.00–00.00)	-0.28	0.09	-3.10	<0.01**
east	−0.17	0.10	−1.82	0.07
south	−0.57	0.08	−6.81	<0.01**
west	−0.13	0.08	−1.56	0.12
east : morning	−0.63	0.23	−2.72	<0.01**
east : afternoon	−1.33	0.29	−4.60	<0.01**
east : evening	0.71	0.27	2.63	<0.01**
south : morning	−1.76	0.21	−8.59	<0.01**
south : afternoon	−3.11	0.25	−12.48	<0.01**
south : evening	−0.30	0.24	−1.28	0.20
west : morning	−1.53	0.19	−7.93	<0.01**
west : afternoon	−1.59	0.24	−6.72	<0.01**
west : evening	0.43	0.23	1.88	0.06
atm.T : morning	1.56	0.38	4.10	<0.01**
atm.T : afternoon	−0.48	0.48	−1.00	0.31
atm.T : evening	1.60	0.44	3.59	<0.01**
(*b*) final best model : T∼1+atm.T+atm.T^2^+atm.T^3^+time of day+nest treatment+nest treatment : time of day+ atm.T : time of day				
intercept	15.25	0.05	284.80	<0.01**
atm.T	11.54	0.23	50.74	<0.01**
atm.T^2^	0.28	0.36	0.77	0.44
atm.T^3^	−2.48	0.44	−5.70	<0.01**
morning (06.00–12.00)	0.76	0.07	11.35	<0.01**
afternoon (12.00–18.00) evening	1.30	0.11	12.00	<0.01**
evening (18.00–00.00)	−0.97	0.08	−11.89	<0.01**
nest.exp.	0.39	0.06	7.07	<0.01**
nest exp. : morning	0.34	0.13	2.63	<0.01**
nest exp. : afternoon	1.41	0.18	7.80	<0.01**
nest exp. : evening	0.43	0.15	2.85	<0.01**
atm.T : morning	0.67	0.33	2.00	0.04
atm.T : afternoon	−0.56	0.42	−1.34	0.18
atm.T : evening	−1.49	0.37	−3.98	<0.01**

The experimental removal of the roof of experimental natural nests influenced the overall temperature of the nest chamber, which was significantly higher in those without a roof, than in intact nests (table 2*b* and figure 3*b*); however, this difference was most pronounced during the hottest period of the day (‘12.00–18.00’; table 2*b*: without roof=1.41±0.18), with no significant difference in the coolest part of the day (‘00.00–06.00’; table 2*b* and figure 3*b*).

### 3.4 Nest microclimate and the development rate of eggs

**Figure 4.**
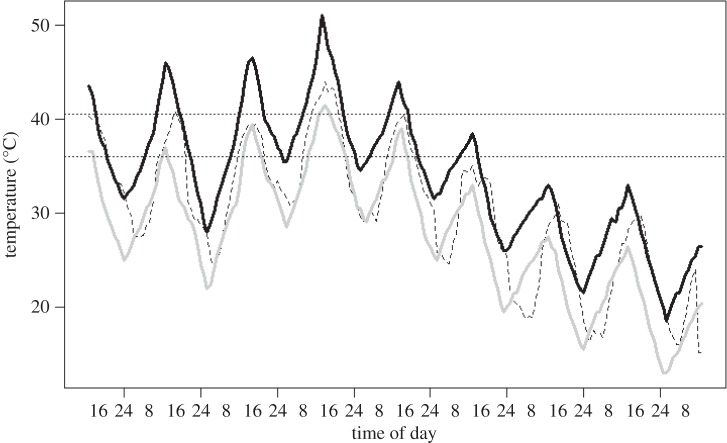
(*a*) Temperatures recorded in hot (solid black line) and cool (solid grey line) experimental nest chambers, in relation to atmospheric temperature (dashed line). The temperature in the chambers was recorded from iButtons in direct contact with experimental eggs, which were subsequently placed back into parental nests and hatched successfully. The horizontal dashed line represents the optimal incubation temperature for avian eggs of 36–40.5°C.

Mean average nest temperatures were 6.1°C higher in experimental hot chambers than in cool chambers (‘hot chamber’ effect=6.1±0.3), and marginally lower in natural nests than in nest-boxes (‘natural nest’ effect=−0.9±0.3). The number of hours per day the nest chambers spent at normal incubation temperatures (36–40°C) varied between hot and cool chambers (table 3*a* and figure 4*a*), with hot chambers spending 2.6 more hours at a normal incubation temperature than cool chambers (‘hot′=5.52±0.83 h; ‘cool′=2.94±0.62 h during the experimental period). There was also considerable variation in the time spent at lethal incubation temperatures (above 40.5°C) between hot and cool chambers (table 3*b* and figure 4*a*), with the hot chambers exceeding this temperature for 3.7 more hours than cool chambers (hot=4.00±0.74 h; cool=0.29±0.21 h). In total, the temperature in the hot chambers exceeded the ‘lethal’ temperature of 40.5°C for 18% of the duration of the experimental period (35 out of 193 hours), and peaked at a temperature of 51°C (figure 4*a*).

**Table 3.** (*a*) Best hurdle model for the number of hours in the day during which experimental chambers (‘hot’ and ‘cool’) were at normal incubation temperatures (36–40°C). (*b*) Best hurdle model for the number of hours in the day during which experimental chambers (‘hot’ and ‘cool’) were at lethal incubation temperatures (above 45°C). (In both analyses, nest type (‘nest-boxes’ and ‘natural nests’) did not feature as a significant predictor. All factors were binarized by splitting them into dummy variables (0,1) and were centred by subtraction of their mean value; because *n* levels can be coded using *n*−1 dummy variables, the ‘cool chamber’ effect is specified by negative values on the remaining dummy variable (‘hot chambers’, corresponding to 0 s in the uncentred dummy variable). ‘Asterisk’ and ‘double asterisks’ indicate significant (*p*<0.05) and highly significant (*p*<0.01) predictors, respectively.)

predictor	estimate	s.e.	*z*-value	*p*
(*a*) hurdle model: time normal∼ sun_C | sun_C				
count model (truncated Poisson with log link)				
intercept	1.60	0.14	11.13	<0.01**
hot chambers	0.54	0.18	3.06	<0.01**
zero hurdle model (binomial with logit link)				
intercept	0.36	0.49	0.72	0.47
hot chamber	0.25	0.71	0.35	0.72
(*b*) hurdle model: time lethal∼ sun_C | sun_C				
count model (truncated Poisson with log link)				
intercept	1.60	0.45	3.52	<0.01**
hot chambers	0.42	0.47	0.89	0.37
zero hurdle model (binomial with logit link)				
intercept	−2.77	1.03	−2.69	<0.01**
hot chambers	2.89	1.14	2.54	0.01*

All of the experimentally treated eggs that had been placed in the experimental chambers hatched successfully following their return to the parental nest, for the period of parental incubation (including all of those subjected to nest temperatures as high as 51°C). This included all of the 17 eggs that were placed into one of the hot chambers. The average developmental time (number of days to hatching from the start of parental incubation) was 13 days (range=12–14) and was shorter for eggs that had been placed in ‘hot chambers’ (mean=12.8±0.11 s.e. versus 13.2±0.10 s.e. days in cool chambers; hot chamber effect=−0.44±0.11) and eggs laid early in the laying sequence also developed and hatched sooner (egg sequence number linear estimate=0.5±0.11): predicted hatching time was 0.4 days shorter for eggs in the ‘hot’ chambers, and 0.9 days shorter for first laid eggs than fifth laid eggs. The time to hatching was similar for eggs placed in the temperature chambers constituted by either the natural nests or the nest-boxes, and none of the interaction terms considered were significant (see the electronic supplementary material, table S3*d*).

## 4. Discussion

We have demonstrated that high temperatures in the nest during the pre-incubation egg-laying period result in significant embryonic development prior to the initiation of parental incubation. Our experimental design, which excluded parents from attending the eggs during the laying period, means that we can attribute this difference to ‘ambient incubation’ and exclude parental incubation or other forms of care. This finding is likely to be of broad significance, because many avian species breed in regions of the world and at times of the year during which atmospheric temperatures frequently exceed the temperature at which their unattended eggs begin to develop (assuming a similar relationship between atmospheric and nest temperature in other species). Furthermore, our data suggest that at a site climatically typical of inland Australia, zebra finches will be regularly exposed to atmospheric temperatures of more than 36°C across six months of the year, when breeding is likely to occur.

Ambient incubation may accrue both benefits and costs to the breeding pair. Atmospheric temperatures that are in the ideal range of incubation will reduce the required time and energetic demand on parents to transfer heat to eggs [20,33,34], and allow adults to forage for longer periods while leaving the nest unattended [9,21,35]. Reduced energy expenditure during incubation, and increased foraging time for parents during the incubation period may positively affect parental condition allowing them to forage more effectively while later feeding nestlings, leading to improved nestling body condition [36].

However, we demonstrated that eggs laid early in the laying sequence (and hence potentially exposed for a longer period to high ambient temperatures before the start of parental incubation), hatched significantly earlier than later laid eggs. Therefore, all else being equal, the hatching asynchrony across a clutch is likely to increase as atmospheric conditions drive the nest temperature towards normal incubation temperatures for longer periods of time. Increased hatching asynchrony is likely to have important fitness consequences. For example, because zebra finch parents passively allocate food across their brood rather than preferentially feeding the smallest nestlings [37], the oldest and largest nestlings usually outcompete their younger, smaller siblings for parentally provided food. As a consequence, variance in quality (e.g. body size, condition) within the brood may be exacerbated and increase the incidence of offspring mortality [18,19].

Given that nest temperatures were not significantly cooler than atmospheric temperatures, and indeed consistently slightly hotter, eggs may also be vulnerable to extreme high temperatures. Previous work in other species suggesting a decrease in viability after periods at high ambient temperature [11], and the widely reported finding that prolonged exposure to temperatures over 40.5°C is lethal to developing avian embryos [16,17]. This is clearly a likely possibility for zebra finches, and other birds sharing the same environment, because inland Australia is subject to periods of extreme heat (between 41 and 46.8°C), particularly in the height of summer, but also in the spring months when the study population typically breeds (October and November; [22]). Although we did not directly measure egg temperatures, we measured the temperature of an iButton that was in direct contact with eggs, finding that temperatures exceeded the lethal threshold for almost 20% of the time during which experimental eggs were treated in the hot chamber. The most extreme hot period was an 11 h stretch in the middle of the trial when recorded temperatures in the hot chambers exceeded 40.5°C, and peaked at 51°C. However, despite these high nest temperatures, all of the 17 eggs that were subjected to the high ambient conditions in the hot chamber successfully hatched after being returned to their parent’s nest and incubated by their parents for the normal incubation period. This suggests that zebra finch eggs may be somewhat more resilient to heat exposure than the eggs of the other species on which the 40.5°C ‘lethal’ threshold has been based [16,17]. The extent of heat tolerance in zebra finch eggs remains to be tested more comprehensively, as well as examining the mechanisms that confer resilience against both a heat load and an increased rate of water loss (e.g. thicker shells [38]).

To mitigate the possible negative effects of high atmospheric temperatures on the development of their embryos and offspring, birds may select nesting sites that are buffered from extreme atmospheric temperatures and the radiant heat of the sun [39,40]. The architecture and orientation of the nests can also be adapted to local climatic conditions to confer thermoregulatory benefits [17,21,41,42]. We demonstrated that zebra finch nest characteristics significantly affect their internal temperature. First, although all zebra finch nests always have a roof, we showed by experimental removal that this aspect of architecture significantly decreases the temperature in the nest chamber, particularly during the hottest part of the day. This suggests that the roof of the zebra finch nest does act to reduce the effect of solar radiation on nest contents. In intact natural nests, temperature in the internal chamber was significantly cooler than at sampled points in the habitat: on average, temperature within nest chambers was 3.7°C cooler than outside during the hottest part of the day (‘12.00–18.00’), but only 1.3° cooler in the evening-night (‘18.00–00.00’). This effect was most likely due to the blocking of direct sunlight, and our data suggest that this may be the primary function of the roof (although presumably it also helps to conceal the nest from predators). Second, our study also identified significant variation in the extent to which the temperature within natural nest chambers reflected changes in atmospheric temperature. In particular, nests that opened with a northerly aspect were significantly hotter during daytime (when the sun is at its strongest), than nests oriented in other directions. Thus, birds could exert some control on the internal temperature of their nests by orienting them in particular directions. While the intrusion of sunlight directly into the nest chamber is best avoided during the hotter periods of the year, earlier in spring and throughout winter birds may instead benefit from the ‘warming’ effect of radiant sunlight in the nest chamber. The extent to which nest orientation may vary across a season with increasing atmospheric temperatures is an open question, and one that is worth investigating given the effects that we have demonstrated here. An earlier study of an open-nesting desert bird found that the positioning of nests varied over the season to reduce solar exposure, but that traded off with predation risk [43].

Our study suggests that radiant sunlight is a significant determinant of the temperatures observed within nest chambers, but an important caveat is that we focused on nests that were not attended by adults. Adults typically do not attend the nest frequently during the laying period in the wild (less than 5% of the day is spent in the nest during this period [23]), but in other avian species living in hot environments a number of additional behavioural and physiological adaptations linked to parental care may contribute to prevent eggs from over-heating. For example, in many open-nesting species, parents stand over the clutch, thereby shading eggs from direct sunlight [40], and rarely leave eggs exposed to solar radiation during the hottest part of the day [44]. In two species of desert-dwelling bird, incubating adults lower the egg temperature by reducing their core body temperature through evaporative cooling [45], directly absorbing heat from eggs that are above 38°C [46,47]. In the study by Arieli *et al.* [47], egg temperatures were maintained at under 42°C, despite the nest reaching 44.9°C and the atmospheric temperature being maintained experimentally at 60°C for over an hour on multiple days. These previous studies [46,47] demonstrating the ability of parents to reduce their body temperature, and subsequently egg temperatures are both focused on *Columbiformes* (doves and pigeons) and we are not aware of any similar demonstrations in *Passeriformes* (the family to which the zebra finch belongs). The zebra finch and its eggs are much smaller, affecting the thermodynamics significantly with respect to the rate of heating and cooling. Both phylogeny and the relative size of the species may affect the degree to which the egg cooling adaptations seen in these earlier studies might be a plausible mechanism for the zebra finch, and indeed species from other avian families inhabiting similarly hot environments. Recent work on a number of desert dwelling birds has revealed an interesting degree of plasticity in the degree to which species vary their basal metabolic rate [48] and their internal body temperature [49], in an effort to cope with the challenges of living in a hot and dry environment. In the latter study of an African passerine (the white-browed sparrow weaver, *Plocepasser mahali*), it was found that during heat stress, adult body temperatures increased by over 2°C over normal set points (hyperthermia) and tolerated the additional heat load to reduce the physiological costs of maintaining a lower temperature [49]. To date, we do not know the extent to which the zebra finch and perhaps its eggs are able to use such strategies in the hotter periods of the breeding season.

Being able to cool eggs through evaporative cooling to withstand the highest temperatures found in our study (inside the nest chamber) would require a dramatic increase (more than 80%) in daily water intake for a passerine bird the size of a zebra finch [50,51]. In arid zone birds, finding additional water for this purpose may often be a challenge [52]. Perhaps tellingly at our study site, the largest aggregations of nests were only found within a kilometre of water, despite suitable patches of vegetation elsewhere [22,25]. In more moist tropical environments, the effectiveness of evaporative cooling may be limited owing to the level of humidity affecting evaporative processes [52]. The reliance on any water-based strategy to cool themselves or their eggs can probably be reduced by adults’ judicious attention to nest architecture and positioning. A well-built nest with a thick roof, in a shaded position and oriented to reduce the impact of solar radiation through the nest entrance will all reduce the internal nest temperature.

Under the increased likelihood of ambient incubation in hot conditions, another distinct possibility is that parents are able to adaptively adjust their investment in eggs (both pre-laying and post-laying), ameliorating the degree to which hatching asynchrony is affected by ambient incubation [53]. This idea is consistent with a recent finding in this species and population that development time varied with laying order [54]. The experimental design of the aforementioned study reveals that this effect was driven by intrinsic properties of the eggs, presumably caused by sequential variation in the allocation of nutrients and/or hormones across the laying sequence [55].

In the light of the hot temperatures that we have observed in nests, and the effects we have observed on the development time of embryos, we believe future research should explore the consequences of ambient incubation for hatching asynchrony, offspring development and adult condition across other avian species occurring in this and other environments around the world. Similarly, the fitness consequences of high atmospheric temperatures, approaching or exceeding lethal thresholds for embryonic, and nestling development, deserve further investigation [13–15]. The extent to which increasing average global temperatures and incidences of heat waves [56] are likely to exacerbate these environmental effects should be considered among the possible threats of a changing climate to tropical and subtropical bird species [17]. In this respect, another important issue that future work needs to address is the extent to which different species may be capable of adapting to climate change, and how behavioural and physiological phenotypic plasticity may contribute to the speed of such adaptations [48,49,53].

## Supplementary Material

R script

## Supplementary Material

additional tables for the GLMM analyses

## Data accessibility

Data available from the Dryad Digital Repository: http://dx.doi.org/10.5061/dryad.7vf87.
