# Supplementary material for: High atmospheric temperatures and ‘ambient incubation’ drive embryonic development and lead to earlier hatching in a passerine bird
Source: R Soc Open Sci. 2016 Feb 3;3(2):150371. doi: 10.1098/rsos.150371 (PMC4785966; doi:10.1098/rsos.150371)
Supplement: R script [file rsos150371supp1.doc]

**# 1) LOCATION**

**setwd("…") # enter file path**

library (nlme)

library(car)

library(AICcmodavg)

data<-read.table('**location.csv**', header=T, sep=';')

attach(data)

date.ID<-date.num

morning<-levels(time.cat)[4]

afternoon<-levels(time.cat)[2]

evening<-levels(time.cat)[3]

night<-levels(time.cat)[1]

morning<-recode(time.cat, "morning=1; else=0 ", as.numeric.result=T, as.factor.result=F )

afternoon<-recode(time.cat, "afternoon=1; else=0 ", as.numeric.result=T, as.factor.result=F )

evening<-recode(time.cat, "evening=1; else=0 ", as.numeric.result=T, as.factor.result=F )

morning_C<-morning-mean(morning)

afternoon_C<-afternoon-mean(afternoon)

evening_C<-evening-mean(evening)

Natural.nest<-recode(Nest.type, " 'Natural nest'=1; else=0 ", as.numeric.result=T, as.factor.result=F )

Natural.nest_C<-Natural.nest-mean(Natural.nest)

Nestbox<-recode(Nest.type, " 'Nestbox'=1; else=0 ", as.numeric.result=T, as.factor.result=F ); Nestbox_C<-Nestbox-mean(Nestbox)

atm.T_C<- (atm.T- mean(atm.T, na.rm=T))/ (2*sd(atm.T, na.rm=T))

**# Models**

**model1**<- gls( Temperature~ 1 + atm.T_C+ I(atm.T_C^2)+ I(atm.T_C^3) + Natural.nest_C+Nestbox_C+ Natural.nest_C:atm.T_C+ Nestbox_C:atm.T_C , correlation=corCompSymm(form=~1|location.ID), weights=varFixed(~atm.T_C) )

**AICc**(model1)

**model2**<- gls( Temperature~ 1 + atm.T_C+ I(atm.T_C^2)+ I(atm.T_C^3)+ Natural.nest_C+Nestbox_C+ Natural.nest_C:atm.T_C+ Nestbox_C:atm.T_C , correlation=corCompSymm(form=~1|location.ID), weights=varComb(varFixed(~atm.T_C), varIdent(~1|Natural.nest_C), varIdent(1|Nestbox_C) ) )

**AICc**(model2)

**model3**<- gls( Temperature~ 1 + atm.T_C+ I(atm.T_C^2)+I(atm.T_C^3)**+**morning_C+ afternoon_C+evening_C+Natural.nest_C+Nestbox_C+ Natural.nest_C:atm.T_C+ Nestbox_C:atm.T_C , correlation=corCompSymm(form=~1|location.ID), weights=varFixed(~atm.T_C) )

**AICc**(model3)

**model4**<- gls( Temperature~ 1 + atm.T_C+ I(atm.T_C^2)+I(atm.T_C^3)+morning_C+ afternoon_C+evening_C+Natural.nest_C+Nestbox_C+ Natural.nest_C:atm.T_C+ Nestbox_C:atm.T_C +morning_C:Natural.nest_C+ afternoon_C:Natural.nest_C+evening_C:Natural.nest_C+morning_C:Nestbox_C+ afternoon_C:Nestbox_C+evening_C:Nestbox_C, correlation=corCompSymm(form=~1|location.ID), weights=varFixed(~atm.T_C) )

**AICc**(model4)

**model5**<- gls( Temperature~ 1 + atm.T_C+ I(atm.T_C^2)+I(atm.T_C^3)+morning_C+ afternoon_C+evening_C+Natural.nest_C+Nestbox_C+morning_C:Natural.nest_C+ afternoon_C:Natural.nest_C+evening_C:Natural.nest_C+morning_C:Nestbox_C+ afternoon_C:Nestbox_C+evening_C:Nestbox_C, correlation=corCompSymm(form=~1|location.ID), weights=varComb(varFixed(~atm.T_C), varIdent(~1|Natural.nest_C), varIdent(1|Nestbox_C) ) )

**AICc**(model5)

summary(model5)

intervals(model5)

**#Figures**

# location

newdata<-data.frame (atm.T_C= rep(0,3), Natural.nest_C= c( max(Natural.nest_C), min(Natural.nest_C), min(Natural.nest_C) ), Nestbox_C= c( min(Nestbox_C), max(Nestbox_C), min(Nestbox_C)) , morning_C=rep(0,3), afternoon_C=rep(0,3), evening_C=rep(0,3) )

y.predicted<- predictSE.gls( mod=model5, newdata=newdata, se.fit=TRUE, print.matrix=T)

error.bars<-function(yv,z,nn) {

xv<-barplot(yv,ylim=c(22,26),names=nn,ylab='Location temperature (°C)', xpd=FALSE, **axis.lty=1, cex.lab=1.2 , cex.names=1.2, font.lab=1.1, cex.axis=1.2** , las=1)

g=(max(xv)-min(xv))/8

for (i in 1:length(xv)) {

lines(c(xv[i],xv[i]),c(yv[i]+z[i],yv[i]-z[i]))

lines(c(xv[i]-g,xv[i]+g),c(yv[i]+z[i], yv[i]+z[i]))

lines(c(xv[i]-g,xv[i]+g),c(yv[i]-z[i], yv[i]-z[i]))} }

labels<-c( 'natural nests', 'nestboxes', 'vegetation')

error.bars(y.predicted$fit, y.predicted$se.fit, labels)

abline(22,0)

# Location * time day _ T=mean x time of the day

newdata.naturalnests<-data.frame (atm.T_C= c(tapply(atm.T_C, time.cat, mean)[1], tapply(atm.T_C, time.cat, mean)[4], tapply(atm.T_C, time.cat, mean)[2],tapply(atm.T_C, time.cat, mean)[3]), Natural.nest_C= rep( max(Natural.nest_C), 4 ), Nestbox_C= rep( min(Nestbox_C), 4) , morning_C=c(min(morning_C), max(morning_C), min(morning_C), min(morning_C)), afternoon_C=c(min(afternoon_C), min(afternoon_C), max(afternoon_C), min(afternoon_C)), evening_C=c(min(evening_C), min(evening_C), min(evening_C), max(evening_C)) )

y.predicted.naturalnests.mean<- predictSE.gls(mod= model5, newdata=newdata.naturalnests, se.fit=T, print.matrix=T)

newdata.nestboxes<-data.frame (atm.T_C= c(tapply(atm.T_C, time.cat, mean)[1], tapply(atm.T_C, time.cat, mean)[4], tapply(atm.T_C, time.cat, mean)[2],tapply(atm.T_C, time.cat, mean)[3]), Natural.nest_C= rep( min(Natural.nest_C), 4 ), Nestbox_C= rep( max(Nestbox_C), 4) , morning_C=c(min(morning_C), max(morning_C), min(morning_C), min(morning_C)), afternoon_C=c(min(afternoon_C), min(afternoon_C), max(afternoon_C), min(afternoon_C)), evening_C=c(min(evening_C), min(evening_C), min(evening_C), max(evening_C)) )

y.predicted.nestboxes.mean<- predictSE.gls( mod=model5, newdata=newdata.nestboxes, se.fit=T, print.matrix=T)

newdata.vegetation<-data.frame (atm.T_C= c(tapply(atm.T_C, time.cat, mean)[1], tapply(atm.T_C, time.cat, mean)[4], tapply(atm.T_C, time.cat, mean)[2],tapply(atm.T_C, time.cat, mean)[3]), Natural.nest_C= rep( min(Natural.nest_C), 4 ), Nestbox_C= rep( min(Nestbox_C), 4) , morning_C=c(min(morning_C), max(morning_C), min(morning_C), min(morning_C)), afternoon_C=c(min(afternoon_C), min(afternoon_C), max(afternoon_C), min(afternoon_C)), evening_C=c(min(evening_C), min(evening_C), min(evening_C), max(evening_C)) )

y.predicted.vegetation.mean<- predictSE.gls( mod=model5, newdata=newdata.vegetation, se.fit=T,print.matrix=T)

# Location * time day _T=max x time of the day

newdata.naturalnests<-data.frame (atm.T_C= c(tapply(atm.T_C, time.cat, max)[1], tapply(atm.T_C, time.cat, max)[4], tapply(atm.T_C, time.cat, max)[2],tapply(atm.T_C, time.cat, max)[3]), Natural.nest_C= rep( max(Natural.nest_C), 4 ), Nestbox_C= rep( min(Nestbox_C), 4) , morning_C=c(min(morning_C), max(morning_C), min(morning_C), min(morning_C)), afternoon_C=c(min(afternoon_C), min(afternoon_C), max(afternoon_C), min(afternoon_C)), evening_C=c(min(evening_C), min(evening_C), min(evening_C), max(evening_C)) )

y.predicted.naturalnests.max<- predictSE.gls( mod=model5, newdata=newdata.naturalnests, se.fit=T, print.matrix=T)

newdata.nestboxes<-data.frame (atm.T_C= c(tapply(atm.T_C, time.cat, max)[1], tapply(atm.T_C, time.cat, max)[4], tapply(atm.T_C, time.cat, max)[2],tapply(atm.T_C, time.cat, max)[3]), Natural.nest_C= rep( min(Natural.nest_C), 4 ), Nestbox_C= rep( max(Nestbox_C), 4) , morning_C=c(min(morning_C), max(morning_C), min(morning_C), min(morning_C)), afternoon_C=c(min(afternoon_C), min(afternoon_C), max(afternoon_C), min(afternoon_C)), evening_C=c(min(evening_C), min(evening_C), min(evening_C), max(evening_C)) )

y.predicted.nestboxes.max<- predictSE.gls( mod=model5, newdata=newdata.nestboxes, se.fit=T, print.matrix=T)

newdata.vegetation<-data.frame (atm.T_C= c(tapply(atm.T_C, time.cat, max)[1], tapply(atm.T_C, time.cat, max)[4], tapply(atm.T_C, time.cat, max)[2],tapply(atm.T_C, time.cat, max)[3]), Natural.nest_C= rep( min(Natural.nest_C), 4 ), Nestbox_C= rep( min(Nestbox_C), 4) , morning_C=c(min(morning_C), max(morning_C), min(morning_C), min(morning_C)), afternoon_C=c(min(afternoon_C), min(afternoon_C), max(afternoon_C), min(afternoon_C)), evening_C=c(min(evening_C), min(evening_C), min(evening_C), max(evening_C)) )

y.predicted.vegetation.max<- predictSE.gls( model5, newdata=newdata.vegetation, se.fit=T, print.matrix=T)

# Location * time day – T_min x time of the day

newdata.naturalnests<-data.frame (atm.T_C= c(tapply(atm.T_C, time.cat, min)[1], tapply(atm.T_C, time.cat, min)[4], tapply(atm.T_C, time.cat, min)[2],tapply(atm.T_C, time.cat, min)[3]), Natural.nest_C= rep( max(Natural.nest_C), 4 ), Nestbox_C= rep( min(Nestbox_C), 4) , morning_C=c(min(morning_C), max(morning_C), min(morning_C), min(morning_C)), afternoon_C=c(min(afternoon_C), min(afternoon_C), max(afternoon_C), min(afternoon_C)), evening_C=c(min(evening_C), min(evening_C), min(evening_C), max(evening_C)) )

y.predicted.naturalnests.min<- predictSE.gls( mod=model5, newdata=newdata.naturalnests, se.fit=T, print.matrix=T)

newdata.nestboxes<-data.frame (atm.T_C= c(tapply(atm.T_C, time.cat, min)[1], tapply(atm.T_C, time.cat, min)[4], tapply(atm.T_C, time.cat, min)[2],tapply(atm.T_C, time.cat, min)[3]), Natural.nest_C= rep( min(Natural.nest_C), 4 ), Nestbox_C= rep( max(Nestbox_C), 4) , morning_C=c(min(morning_C), max(morning_C), min(morning_C), min(morning_C)), afternoon_C=c(min(afternoon_C), min(afternoon_C), max(afternoon_C), min(afternoon_C)), evening_C=c(min(evening_C), min(evening_C), min(evening_C), max(evening_C)) )

y.predicted.nestboxes.min<- predictSE.gls(mod= model5, newdata=newdata.nestboxes, se.fit=T, print.matrix=T)

newdata.vegetation<-data.frame (atm.T_C= c(tapply(atm.T_C, time.cat, min)[1], tapply(atm.T_C, time.cat, min)[4], tapply(atm.T_C, time.cat, min)[2],tapply(atm.T_C, time.cat, min)[3]), Natural.nest_C= rep( min(Natural.nest_C), 4 ), Nestbox_C= rep( min(Nestbox_C), 4) , morning_C=c(min(morning_C), max(morning_C), min(morning_C), min(morning_C)), afternoon_C=c(min(afternoon_C), min(afternoon_C), max(afternoon_C), min(afternoon_C)), evening_C=c(min(evening_C), min(evening_C), min(evening_C), max(evening_C)) )

y.predicted.vegetation.min<- predictSE.gls( mod=model5, newdata=newdata.vegetation, se.fit=T, print.matrix=T)

space<-c(-40,-40)

predicted.mean<-c( c(y.predicted.naturalnests.mean[1,1], y.predicted.nestboxes.mean[1,1], y.predicted.vegetation.mean[1,1]), space, c(y.predicted.naturalnests.mean[2,1], y.predicted.nestboxes.mean[2,1], y.predicted.vegetation.mean[2,1]), space, c(y.predicted.naturalnests.mean[3,1], y.predicted.nestboxes.mean[3,1], y.predicted.vegetation.mean[3,1]), space, c(y.predicted.naturalnests.mean[4,1], y.predicted.nestboxes.mean[4,1], y.predicted.vegetation.mean[4,1]))

predicted.SE<-c( c(y.predicted.naturalnests.mean[1,2], y.predicted.nestboxes.mean[1,2], y.predicted.vegetation.mean[1,2]),space, c(y.predicted.naturalnests.mean[2,2], y.predicted.nestboxes.mean[2,2], y.predicted.vegetation.mean[2,2]),space, c(y.predicted.naturalnests.mean[3,2], y.predicted.nestboxes.mean[3,2], y.predicted.vegetation.mean[3,2]), space, c(y.predicted.naturalnests.mean[4,2], y.predicted.nestboxes.mean[4,2], y.predicted.vegetation.mean[4,2]))

predicted.lower<- predicted.mean - predicted.SE

predicted.upper<- predicted.mean + predicted.SE

plot(predicted.mean, ylim=c(0,50) , xlab='', xaxt='n', col='black', pch=c(1,0,2,3,3,1,0,2,3,3,1,0,2,3,3,1,0,2), ylab='',yaxs="i", axis.lty=1, cex.lab=1.2 , cex.names=1.2, font.lab=1.1, cex.axis=1.2 , las=1

)

arrows( seq(1:18), predicted.lower, seq(1:18), predicted.upper, code=3, angle=90, length=0.1, col='black')

par(new=T)

predicted.mean<-c( c(y.predicted.naturalnests.max[1,1], y.predicted.nestboxes.max[1,1], y.predicted.vegetation.max[1,1]), space, c(y.predicted.naturalnests.max[2,1], y.predicted.nestboxes.max[2,1], y.predicted.vegetation.max[2,1]), space, c(y.predicted.naturalnests.max[3,1], y.predicted.nestboxes.max[3,1], y.predicted.vegetation.max[3,1]), space, c(y.predicted.naturalnests.max[4,1], y.predicted.nestboxes.max[4,1], y.predicted.vegetation.max[4,1]))

predicted.SE<-c( c(y.predicted.naturalnests.max[1,2], y.predicted.nestboxes.max[1,2], y.predicted.vegetation.max[1,2]),space, c(y.predicted.naturalnests.max[2,2], y.predicted.nestboxes.max[2,2], y.predicted.vegetation.max[2,2]),space, c(y.predicted.naturalnests.max[3,2], y.predicted.nestboxes.max[3,2], y.predicted.vegetation.max[3,2]), space, c(y.predicted.naturalnests.max[4,2], y.predicted.nestboxes.max[4,2], y.predicted.vegetation.max[4,2]))

predicted.lower<- predicted.mean - predicted.SE

predicted.upper<- predicted.mean + predicted.SE

plot(predicted.mean, ylim=c(0,50) , xlab='', xaxt='n', col='dark grey', pch=c(1,0,2,3,3,1,0,2,3,3,1,0,2,3,3,1,0,2), ylab='',yaxs="i", axis.lty=1, cex.lab=1.2 , cex.names=1.2, font.lab=1.1, cex.axis=1.2 , las=1 )

arrows( seq(1:18), predicted.lower, seq(1:18), predicted.upper, code=3, angle=90, length=0.1, col='dark grey')

par(new=T)

predicted.mean<-c( c(y.predicted.naturalnests.min[1,1], y.predicted.nestboxes.min[1,1], y.predicted.vegetation.min[1,1]), space, c(y.predicted.naturalnests.min[2,1], y.predicted.nestboxes.min[2,1], y.predicted.vegetation.min[2,1]), space, c(y.predicted.naturalnests.min[3,1], y.predicted.nestboxes.min[3,1], y.predicted.vegetation.min[3,1]), space, c(y.predicted.naturalnests.min[4,1], y.predicted.nestboxes.min[4,1], y.predicted.vegetation.min[4,1]))

predicted.SE<-c( c(y.predicted.naturalnests.min[1,2], y.predicted.nestboxes.min[1,2], y.predicted.vegetation.min[1,2]),space, c(y.predicted.naturalnests.min[2,2], y.predicted.nestboxes.min[2,2], y.predicted.vegetation.min[2,2]),space, c(y.predicted.naturalnests.min[3,2], y.predicted.nestboxes.min[3,2], y.predicted.vegetation.min[3,2]), space, c(y.predicted.naturalnests.min[4,2], y.predicted.nestboxes.min[4,2], y.predicted.vegetation.min[4,2]))

predicted.lower<- predicted.mean - predicted.SE

predicted.upper<- predicted.mean + predicted.SE

plot(predicted.mean, ylim=c(0,50) , xlab='', xaxt='n', col='dark grey', pch=c(1,0,2,3,3,1,0,2,3,3,1,0,2,3,3,1,0,2), ylab='Temperature location (°C)',yaxs="i", axis.lty=1, cex.lab=1.2 , cex.names=1.2, font.lab=1.1, cex.axis=1.2 , las=1 )

arrows( seq(1:18), predicted.lower, seq(1:18), predicted.upper, code=3, angle=90, length=0.1, col='dark grey')

abline(40.5, 0, lty=3); abline(36, 0, lty=3)

abline(0,0)

axis(1, labels=c('0:00-6:00','6:00-12:00','12:00-18:00', '18:00-24:00'), at=c(2,7,12,17), tick=FALSE, cex=1.2)

# atm Temp * location _ afternoon

data2<-cbind(data, atm.T_C)

data2<-subset(data2, time.cat=='12:00-18:00')

atm.T_C.afternoon<- data2$atm.T_C

newdata<-data.frame (atm.T_C= seq(-1.16,1.1,0.1) , morning_C=rep(min(morning_C), 23), afternoon_C=rep(max(afternoon_C), 23), evening_C=rep(min(evening_C), 23), Natural.nest_C= rep( max(Natural.nest_C), 23 ), Nestbox_C= rep( min(Nestbox_C), 23 ) )

y.predicted.naturalnests<- predictSE.gls( mod=model5, newdata=newdata, se.fit=TRUE, print.matrix=T)

y.predicted.mean<-y.predicted.naturalnests[,1]

error<-y.predicted.naturalnests[,2]

CI_upper<-(y.predicted.mean+ error)

CI_lower<-(y.predicted.mean- error)

data.naturalnests<-subset(data2, Nest.type=='Natural nest')

# plot (data.naturalnests$atm.T, jitter(data.naturalnests$Temperature),xlab=' ', ylab= 'Temperature location' , xlim=c(5,50) , ylim=c(5,50), cex=0.3, col='red' )

par(new=T)

x<- mean(atm.T, na.rm=T)+seq(-1.16,1.1,0.1)*(2*sd(atm.T, na.rm=T))

plot (x, y.predicted.mean, xlab=' ', ylab= ' ', xlim=c(10,45) , ylim=c(10,45), cex=0.0, **axis.lty=1, cex.lab=1.2 , cex.names=1.2, font.lab=1.1, cex.axis=1.2** , las=1)

abline (36,0, col='black', lty=3); abline (40.5,0, col='black', lty=3)

lines( x, y.predicted.mean )

lines(loess(CI_upper~ x ) )

lines(loess(CI_lower~ x ) )

par(new=TRUE)

newdata<-data.frame (atm.T_C= seq(-1.16,1.1,0.1) , morning_C=rep(min(morning_C), 23), afternoon_C=rep(max(afternoon_C), 23), evening_C=rep(min(evening_C), 23), Natural.nest_C= rep( min(Natural.nest_C), 23 ), Nestbox_C= rep( max(Nestbox_C), 23 ) )

y.predicted.nestboxes<- predictSE.gls( mod=model5, newdata=newdata, se.fit=TRUE, print.matrix=T)

y.predicted.mean<-y.predicted.nestboxes[,1]

error<-y.predicted.nestboxes[,2]

CI_upper<-(y.predicted.mean+ error)

CI_lower<-(y.predicted.mean- error)

data.nestboxes<-subset(data2, Nest.type=='Nestbox')

# plot (data.nestboxes$atm.T, jitter(data.nestboxes$Temperature),xlab=' ', ylab= 'Temperature location' , xlim=c(5,50) , ylim=c(5,50), cex=0.3, col='blue' )

par(new=T)

x<- mean(atm.T, na.rm=T)+seq(-1.16,1.1,0.1)*(2*sd(atm.T, na.rm=T))

plot (x, y.predicted.mean, xlab='', ylab= '', xlim=c(10,45) , ylim=c(10,45), **axis.lty=1, cex.lab=1.2 , cex.names=1.2, font.lab=1.1, cex.axis=1.2** , las=1 ,cex=0.0)

# abline(0,1, col='black')

lines( x, y.predicted.mean , lty=2)

lines(loess(CI_upper~ x ) , lty=2 )

lines(loess(CI_lower~ x ), lty=2 )

par(new=T)

newdata<-data.frame (atm.T_C= seq(-1.16,1.1,0.1) , morning_C=rep(min(morning_C), 23), afternoon_C=rep(max(afternoon_C), 23), evening_C=rep(min(evening_C), 23), Natural.nest_C= rep( min(Natural.nest_C), 23 ), Nestbox_C= rep( min(Nestbox_C), 23 ) )

y.predicted.vegetation<- predictSE.gls( mod=model5, newdata=newdata, se.fit=TRUE, print.matrix=T)

y.predicted.mean<-y.predicted.vegetation[,1]

error<-y.predicted.vegetation[,2]

CI_upper<-(y.predicted.mean+ error)

CI_lower<-(y.predicted.mean- error)

data.vegetation<-subset(data2, Nest.type=='Vegetation')

# plot (data.vegetation $atm.T, jitter(data.vegetation $Temperature),xlab=' ', ylab= 'Temperature location' , xlim=c(5,50) , ylim=c(5,50), cex=0.3, col='dark green' )

par(new=T)

x<- mean(atm.T, na.rm=T)+seq(-1.16,1.1,0.1)*(2*sd(atm.T, na.rm=T))

plot (x, y.predicted.mean, xlab=' Atmospheric temperature (°C)', ylab= 'Location temperature (°C)', xlim=c(10,45) , ylim=c(10,45), cex=0.00 ,**axis.lty=1, cex.lab=1.2 , cex.names=1.2, font.lab=1.1, cex.axis=1.2** , las=1)

lines( x, y.predicted.mean , lty=3 )

lines(loess(CI_upper~ x ) , lty=3)

lines(loess(CI_lower~ x ) , lty=3)

**# 2) NATURAL NESTS – ORIENTATION, HEIGHT**

data<-read.table('natural nests.csv', header=T, sep=';')

attach(data)

library (nlme)

library(car)

library(AICcmodavg)

date.ID<-date.num

Temperature<-as.numeric(as.character(Temperature))

Temperature<-round(Temperature,2)

atm.T_C<- (atm.T- mean(atm.T, na.rm=T))/ (2*sd(atm.T, na.rm=T))

nest.height_C<- (Nest.height -mean(Nest.height, na.rm=T))/ (2*sd(Nest.height, na.rm=T))

N<-recode(Nest.orientation.cat, " 'North'=1; else=0 ", as.numeric.result=T, as.factor.result=F );

N_C<-N-mean(N)

E<-recode(Nest.orientation.cat, " 'East'=1; else=0 ", as.numeric.result=T, as.factor.result=F );

E_C<-E-mean(E)

S<-recode(Nest.orientation.cat, " 'South'=1; else=0 ", as.numeric.result=T, as.factor.result=F );

S_C<-S-mean(S)

W<-recode(Nest.orientation.cat, " 'West'=1; else=0 ", as.numeric.result=T, as.factor.result=F );

W_C<-W-mean(W)

morning<-levels(time.cat)[4]

afternoon<-levels(time.cat)[2]

evening<-levels(time.cat)[3]

night<-levels(time.cat)[1]

morning<-recode(time.cat, "morning=1 ; else=0 ", as.numeric.result=T, as.factor.result=F )

afternoon<-recode(time.cat, "afternoon=1 ; else=0 ", as.numeric.result=T, as.factor.result=F )

evening<-recode(time.cat, "evening=1 ; else=0 ", as.numeric.result=T, as.factor.result=F )

morning_C<-morning-mean(morning)

afternoon_C<-afternoon-mean(afternoon)

evening_C<-evening-mean(evening)

model.0<- gls( Temperature~ 1 + atm.T_C+ I(atm.T_C^2)+ I(atm.T_C^3)+morning_C+ afternoon_C+evening_C+ morning_C:atm.T_C+ afternoon_C:atm.T_C+evening_C:atm.T_C, correlation=corCompSymm(form=~1|Nest.ID + 1|date.ID), weights=varFixed(~atm.T_C) )

AICc(model.0)

model.1<- gls( Temperature~ 1 + atm.T_C+ I(atm.T_C^2)+ I(atm.T_C^3)+morning_C+ afternoon_C+evening_C+ E_C+ S_C+W_C+morning_C:atm.T_C+ afternoon_C:atm.T_C+evening_C:atm.T_C, correlation=corCompSymm(form=~1|Nest.ID + 1|date.ID), weights=varFixed(~atm.T_C) )

AICc(model.1)

model.2<- gls( Temperature~ 1 + atm.T_C+ I(atm.T_C^2)+ I(atm.T_C^3)+morning_C+ afternoon_C+evening_C+ E_C+ S_C +W_C+ E_C:morning_C+ E_C:afternoon_C+ E_C:evening_C+ S_C:morning_C+ S_C:afternoon_C+ S_C:evening_C + W_C:morning_C+ W_C:afternoon_C+ W_C:evening_C+morning_C:atm.T_C+ afternoon_C:atm.T_C+ evening_C:atm.T_C, correlation=corCompSymm(form=~1|Nest.ID + 1|date.ID), weights=varFixed(~atm.T_C) )

AICc(model.2)

model.3<- gls( Temperature~ 1 + atm.T_C+ I(atm.T_C^2)+ I(atm.T_C^3)+morning_C+ afternoon_C+evening_C+ E_C+ S_C+W_C+nest.height_C+ E_C:morning_C+ E_C:afternoon_C++ E_C:evening_C+ S_C:morning_C+ S_C:afternoon_C+ S_C:evening_C + W_C:morning_C+ W_C:afternoon_C+ W_C:evening_C+morning_C:nest.height_C+ afternoon_C:nest.height_C+evening_C:nest.height_C+morning_C:atm.T_C+ afternoon_C:atm.T_C+evening_C:atm.T_C, correlation=corCompSymm(form=~1|Nest.ID + 1|date.ID), weights=varFixed(~atm.T_C) )

AICc(model.3)

model.4<- gls( Temperature~ 1 + atm.T_C+ I(atm.T_C^2)+ I(atm.T_C^3)+morning_C+ afternoon_C+evening_C+ E_C+ S_C+W_C+nest.height_C+ E_C:morning_C+ E_C:afternoon_C++ E_C:evening_C+ S_C:morning_C+ S_C:afternoon_C+ S_C:evening_C + W_C:morning_C+ W_C:afternoon_C+ W_C:evening_C+morning_C:nest.height_C+ afternoon_C:nest.height_C+evening_C:nest.height_C+ atm.T_C:nest.height_C, correlation=corCompSymm(form=~1|Nest.ID + 1|date.ID), weights=varFixed(~atm.T_C) )

AICc(model.4)

model.5<- gls( Temperature~ 1 + atm.T_C+ I(atm.T_C^2)+ I(atm.T_C^3)+morning_C+ afternoon_C+evening_C+ E_C+ S_C+W_C+nest.height_C+ E_C:morning_C+ E_C:afternoon_C++ E_C:evening_C+ S_C:morning_C+ S_C:afternoon_C+ S_C:evening_C + W_C:morning_C+ W_C:afternoon_C+ W_C:evening_C+morning_C:nest.height_C+ afternoon_C:nest.height_C+evening_C:nest.height_C+ atm.T_C:E_C+ atm.T_C:S_C+ atm.T_C:W_C, correlation=corCompSymm(form=~1|Nest.ID + 1|date.ID), weights=varFixed(~atm.T_C) )

AICc(model.5)

model.6<- gls( Temperature~ 1 + atm.T_C+ I(atm.T_C^2)+ I(atm.T_C^3)+morning_C+ afternoon_C+evening_C+ E_C+ S_C+W_C+nest.height_C+ E_C:morning_C+ E_C:afternoon_C++ E_C:evening_C+ S_C:morning_C+ S_C:afternoon_C+ S_C:evening_C + W_C:morning_C+ W_C:afternoon_C+ W_C:evening_C+morning_C:atm.T_C+ afternoon_C:atm.T_C+evening_C:atm.T_C, correlation=corCompSymm(form=~1|Nest.ID + 1|date.ID), weights=varFixed(~atm.T_C) )

AICc(model.6)

model.7<- gls( Temperature~ 1 + atm.T_C+ I(atm.T_C^2)+ I(atm.T_C^3)+morning_C+ afternoon_C+evening_C, correlation=corCompSymm(form=~1|Nest.ID + 1|date.ID), weights=varFixed(~atm.T_C) )

AICc(model.7)

model.8<- gls( Temperature~ 1 + atm.T_C+ I(atm.T_C^2)+ I(atm.T_C^3)+morning_C+ afternoon_C+evening_C+ E_C+ S_C +W_C+ E_C:morning_C+ E_C:afternoon_C+ E_C:evening_C+ S_C:morning_C+ S_C:afternoon_C+ S_C:evening_C + W_C:morning_C+ W_C:afternoon_C+ W_C:evening_C, correlation=corCompSymm(form=~1|Nest.ID + 1|date.ID), weights=varFixed(~atm.T_C) )

AICc(model.8)

summary(model.2)

confint(model.2)

**# Figures**

barplot(c(sum(N), sum(E), sum(S), sum(W)), names.arg= c('North', 'East', 'South', 'West'), for and ylab=' No. nests')

**# Orientation * time day _T=mean x time of the day**

newdata.north<-data.frame (atm.T_C= c(tapply(atm.T_C, time.cat, mean)[1], tapply(atm.T_C, time.cat, mean)[4], tapply(atm.T_C, time.cat, mean)[2],tapply(atm.T_C, time.cat, mean)[3]), E_C= rep( min(E_C), 4 ), S_C= rep( min(S_C), 4) ,W_C= rep( min(W_C), 4), morning_C=c(min(morning_C), max(morning_C), min(morning_C), min(morning_C)), afternoon_C=c(min(afternoon_C), min(afternoon_C), max(afternoon_C), min(afternoon_C)), evening_C=c(min(evening_C), min(evening_C), min(evening_C), max(evening_C)) , nest.height_C=rep(0,4) )

y.predicted.north.mean<- predictSE.gls(mod= model.2 ,newdata=newdata.north, se.fit=TRUE, print.matrix=T)

newdata.east<-data.frame (atm.T_C= c(tapply(atm.T_C, time.cat, mean)[1], tapply(atm.T_C, time.cat, mean)[4], tapply(atm.T_C, time.cat, mean)[2],tapply(atm.T_C, time.cat, mean)[3]), E_C= rep( max(E_C), 4 ), S_C= rep( min(S_C), 4) ,W_C= rep( min(W_C), 4), morning_C=c(min(morning_C), max(morning_C), min(morning_C), min(morning_C)), afternoon_C=c(min(afternoon_C), min(afternoon_C), max(afternoon_C), min(afternoon_C)), evening_C=c(min(evening_C), min(evening_C), min(evening_C), max(evening_C)) , nest.height_C=rep(0,4))

y.predicted.east.mean<- predictSE.gls( mod=model.2, newdata=newdata.east, se.fit=TRUE, print.matrix=T)

newdata.south<-data.frame (atm.T_C= c(tapply(atm.T_C, time.cat, mean)[1], tapply(atm.T_C, time.cat, mean)[4], tapply(atm.T_C, time.cat, mean)[2],tapply(atm.T_C, time.cat, mean)[3]), E_C= rep( min(E_C), 4 ), S_C= rep( max(S_C), 4) ,W_C= rep( min(W_C), 4), morning_C=c(min(morning_C), max(morning_C), min(morning_C), min(morning_C)), afternoon_C=c(min(afternoon_C), min(afternoon_C), max(afternoon_C), min(afternoon_C)), evening_C=c(min(evening_C), min(evening_C), min(evening_C), max(evening_C)) , nest.height_C=rep(0,4))

y.predicted.south.mean<- predictSE.gls(mod= model.2 ,newdata=newdata.south, se.fit=TRUE, print.matrix=T)

newdata.west<-data.frame (atm.T_C= c(tapply(atm.T_C, time.cat, mean)[1], tapply(atm.T_C, time.cat, mean)[4], tapply(atm.T_C, time.cat, mean)[2],tapply(atm.T_C, time.cat, mean)[3]), E_C= rep( min(E_C), 4 ), S_C= rep( min(S_C), 4) ,W_C= rep( max(W_C), 4), morning_C=c(min(morning_C), max(morning_C), min(morning_C), min(morning_C)), afternoon_C=c(min(afternoon_C), min(afternoon_C), max(afternoon_C), min(afternoon_C)), evening_C=c(min(evening_C), min(evening_C), min(evening_C), max(evening_C)) , nest.height_C=rep(0,4))

y.predicted.west.mean<- predictSE.gls( mod=model.2 ,newdata=newdata.west, se.fit=TRUE, print.matrix=T)

**# Orientation * time day _ T=max x time of the day**

newdata.north<-data.frame (atm.T_C= c(tapply(atm.T_C, time.cat, max)[1], tapply(atm.T_C, time.cat, max)[4], tapply(atm.T_C, time.cat, max)[2],tapply(atm.T_C, time.cat, max)[3]), E_C= rep( min(E_C), 4 ), S_C= rep( min(S_C), 4) ,W_C= rep( min(W_C), 4), morning_C=c(min(morning_C), max(morning_C), min(morning_C), min(morning_C)), afternoon_C=c(min(afternoon_C), min(afternoon_C), max(afternoon_C), min(afternoon_C)), evening_C=c(min(evening_C), min(evening_C), min(evening_C), max(evening_C)) , nest.height_C=rep(0,4) )

y.predicted.north.max<- predictSE.gls(mod= model.2 ,newdata=newdata.north, se.fit=TRUE, print.matrix=T)

newdata.east<-data.frame (atm.T_C= c(tapply(atm.T_C, time.cat, max)[1], tapply(atm.T_C, time.cat, max)[4], tapply(atm.T_C, time.cat, max)[2],tapply(atm.T_C, time.cat, max)[3]), E_C= rep( max(E_C), 4 ), S_C= rep( min(S_C), 4) ,W_C= rep( min(W_C), 4), morning_C=c(min(morning_C), max(morning_C), min(morning_C), min(morning_C)), afternoon_C=c(min(afternoon_C), min(afternoon_C), max(afternoon_C), min(afternoon_C)), evening_C=c(min(evening_C), min(evening_C), min(evening_C), max(evening_C)) , nest.height_C=rep(0,4))

y.predicted.east.max<- predictSE.gls( mod=model.2, newdata=newdata.east, se.fit=TRUE, print.matrix=T)

newdata.south<-data.frame (atm.T_C= c(tapply(atm.T_C, time.cat, max)[1], tapply(atm.T_C, time.cat, max)[4], tapply(atm.T_C, time.cat, max)[2],tapply(atm.T_C, time.cat, max)[3]), E_C= rep( min(E_C), 4 ), S_C= rep( max(S_C), 4) ,W_C= rep( min(W_C), 4), morning_C=c(min(morning_C), max(morning_C), min(morning_C), min(morning_C)), afternoon_C=c(min(afternoon_C), min(afternoon_C), max(afternoon_C), min(afternoon_C)), evening_C=c(min(evening_C), min(evening_C), min(evening_C), max(evening_C)) , nest.height_C=rep(0,4))

y.predicted.south.max<- predictSE.gls( mod=model.2 ,newdata=newdata.south, se.fit=TRUE, print.matrix=T)

newdata.west<-data.frame (atm.T_C= c(tapply(atm.T_C, time.cat, max)[1], tapply(atm.T_C, time.cat, max)[4], tapply(atm.T_C, time.cat, max)[2],tapply(atm.T_C, time.cat, max)[3]), E_C= rep( min(E_C), 4 ), S_C= rep( min(S_C), 4) ,W_C= rep( max(W_C), 4), morning_C=c(min(morning_C), max(morning_C), min(morning_C), min(morning_C)), afternoon_C=c(min(afternoon_C), min(afternoon_C), max(afternoon_C), min(afternoon_C)), evening_C=c(min(evening_C), min(evening_C), min(evening_C), max(evening_C)) , nest.height_C=rep(0,4))

y.predicted.west.max<- predictSE.gls( mod=model.2 ,newdata=newdata.west, se.fit=TRUE, print.matrix=T)

**# Orientation * time day _ T=min x time of the day**

newdata.north<-data.frame (atm.T_C= c(tapply(atm.T_C, time.cat, min)[1], tapply(atm.T_C, time.cat, min)[4], tapply(atm.T_C, time.cat, min)[2],tapply(atm.T_C, time.cat, min)[3]), E_C= rep( min(E_C), 4 ), S_C= rep( min(S_C), 4) ,W_C= rep( min(W_C), 4), morning_C=c(min(morning_C), max(morning_C), min(morning_C), min(morning_C)), afternoon_C=c(min(afternoon_C), min(afternoon_C), max(afternoon_C), min(afternoon_C)), evening_C=c(min(evening_C), min(evening_C), min(evening_C), max(evening_C)) , nest.height_C=rep(0,4) )

y.predicted.north.min<- predictSE.gls(mod= model.2 ,newdata=newdata.north, se.fit=TRUE, print.matrix=T)

newdata.east<-data.frame (atm.T_C= c(tapply(atm.T_C, time.cat, min)[1], tapply(atm.T_C, time.cat, min)[4], tapply(atm.T_C, time.cat, min)[2],tapply(atm.T_C, time.cat, min)[3]), E_C= rep( max(E_C), 4 ), S_C= rep( min(S_C), 4) ,W_C= rep( min(W_C), 4), morning_C=c(min(morning_C), max(morning_C), min(morning_C), min(morning_C)), afternoon_C=c(min(afternoon_C), min(afternoon_C), max(afternoon_C), min(afternoon_C)), evening_C=c(min(evening_C), min(evening_C), min(evening_C), max(evening_C)) , nest.height_C=rep(0,4))

y.predicted.east.min<- predictSE.gls( mod=model.2, newdata=newdata.east, se.fit=TRUE, print.matrix=T)

newdata.south<-data.frame (atm.T_C= c(tapply(atm.T_C, time.cat, min)[1], tapply(atm.T_C, time.cat, min)[4], tapply(atm.T_C, time.cat, min)[2],tapply(atm.T_C, time.cat, min)[3]), E_C= rep( min(E_C), 4 ), S_C= rep( max(S_C), 4) ,W_C= rep( min(W_C), 4), morning_C=c(min(morning_C), max(morning_C), min(morning_C), min(morning_C)), afternoon_C=c(min(afternoon_C), min(afternoon_C), max(afternoon_C), min(afternoon_C)), evening_C=c(min(evening_C), min(evening_C), min(evening_C), max(evening_C)) , nest.height_C=rep(0,4))

y.predicted.south.min<- predictSE.gls( mod=model.2 ,newdata=newdata.south, se.fit=TRUE, print.matrix=T)

newdata.west<-data.frame (atm.T_C= c(tapply(atm.T_C, time.cat, min)[1], tapply(atm.T_C, time.cat, min)[4], tapply(atm.T_C, time.cat, min)[2],tapply(atm.T_C, time.cat, min)[3]), E_C= rep( min(E_C), 4 ), S_C= rep( min(S_C), 4) ,W_C= rep( max(W_C), 4), morning_C=c(min(morning_C), max(morning_C), min(morning_C), min(morning_C)), afternoon_C=c(min(afternoon_C), min(afternoon_C), max(afternoon_C), min(afternoon_C)), evening_C=c(min(evening_C), min(evening_C), min(evening_C), max(evening_C)) , nest.height_C=rep(0,4))

y.predicted.west.min<- predictSE.gls(mod= model.2 ,newdata=newdata.west, se.fit=TRUE, print.matrix=T)

space<-c(-50,-50)

orientation<-rep(c('N', 'E', 'S', 'W','',''),4)

predicted.mean<-c( c(y.predicted.north.mean[1,1], y.predicted.east.mean[1,1], y.predicted.south.mean[1,1],y.predicted.west.mean[1,1]) , space, c(y.predicted.north.mean[2,1], y.predicted.east.mean[2,1], y.predicted.south.mean[2,1],y.predicted.west.mean[2,1]) , space, c(y.predicted.north.mean[3,1], y.predicted.east.mean[3,1], y.predicted.south.mean[3,1],y.predicted.west.mean[3,1]) , space, c(y.predicted.north.mean[4,1], y.predicted.east.mean[4,1], y.predicted.south.mean[4,1],y.predicted.west.mean[4,1]) )

predicted.SE<-c( c(y.predicted.north.mean[1,2], y.predicted.east.mean[1,2], y.predicted.south.mean[1,2],y.predicted.west.mean[1,2]) , space, c(y.predicted.north.mean[2,2], y.predicted.east.mean[2,2], y.predicted.south.mean[2,2],y.predicted.west.mean[2,2]) , space, c(y.predicted.north.mean[3,2], y.predicted.east.mean[3,2], y.predicted.south.mean[3,2],y.predicted.west.mean[3,2]) , space, c(y.predicted.north.mean[4,2], y.predicted.east.mean[4,2], y.predicted.south.mean[4,2],y.predicted.west.mean[4,2]) )

predicted.lower<- predicted.mean - predicted.SE

predicted.upper<- predicted.mean + predicted.SE

plot(predicted.mean, ylim=c(0,50) , xlab='', col='black', ylab='',yaxs="i", xaxt='n', **axis.lty=1, cex.lab=1.2 , cex.names=1.2, font.lab=1.1, cex.axis=1.2** , las=1 )

text(c( c(y.predicted.north.mean[1,1], y.predicted.east.mean[1,1], y.predicted.south.mean[1,1],y.predicted.west.mean[1,1]) , space, c(y.predicted.north.mean[2,1], y.predicted.east.mean[2,1], y.predicted.south.mean[2,1],y.predicted.west.mean[2,1]) , space, c(y.predicted.north.mean[3,1], y.predicted.east.mean[3,1], y.predicted.south.mean[3,1],y.predicted.west.mean[3,1]) , space, c(y.predicted.north.mean[4,1], y.predicted.east.mean[4,1], y.predicted.south.mean[4,1],y.predicted.west.mean[4,1]) )+3, labels=orientation)

arrows( seq(1:22), predicted.lower, seq(1:22), predicted.upper, code=3, angle=90, length=0.1, col='black')

par(new=T)

predicted.mean*<-*c( c(y.predicted.north.max[1,1], y.predicted.east.max[1,1], y.predicted.south.max[1,1],y.predicted.west.max[1,1]) , space, c(y.predicted.north.max[2,1], y.predicted.east.max[2,1], y.predicted.south.max[2,1],y.predicted.west.max[2,1]) , space, c(y.predicted.north.max[3,1], y.predicted.east.max[3,1], y.predicted.south.max[3,1],y.predicted.west.max[3,1]) , space, c(y.predicted.north.max[4,1], y.predicted.east.max[4,1], y.predicted.south.max[4,1],y.predicted.west.max[4,1]) )

predicted.SE<-c( c(y.predicted.north.max[1,2], y.predicted.east.max[1,2], y.predicted.south.max[1,2],y.predicted.west.max[1,2]) , space, c(y.predicted.north.max[2,2], y.predicted.east.max[2,2], y.predicted.south.max[2,2],y.predicted.west.max[2,2]) , space, c(y.predicted.north.max[3,2], y.predicted.east.max[3,2], y.predicted.south.max[3,2],y.predicted.west.max[3,2]) , space, c(y.predicted.north.max[4,2], y.predicted.east.max[4,2], y.predicted.south.max[4,2],y.predicted.west.max[4,2]) )

predicted.lower<- predicted.mean -predicted.SE

predicted.upper<- predicted.mean+ predicted.SE

plot(predicted.mean, ylim=c(0,50) , xlab='', col='dark grey', ylab='',yaxs="i", xaxt='n', **axis.lty=1, cex.lab=1.2 , cex.names=1.2, font.lab=1.1, cex.axis=1.2** , las=1 )

text(c( c(y.predicted.north.max[1,1], y.predicted.east.max[1,1], y.predicted.south.max[1,1],y.predicted.west.max[1,1]) , space, c(y.predicted.north.max[2,1], y.predicted.east.max[2,1], y.predicted.south.max[2,1],y.predicted.west.max[2,1]) , space, c(y.predicted.north.max[3,1], y.predicted.east.max[3,1], y.predicted.south.max[3,1],y.predicted.west.max[3,1]) , space, c(y.predicted.north.max[4,1], y.predicted.east.max[4,1], y.predicted.south.max[4,1],y.predicted.west.max[4,1]) )+3, labels=orientation)

arrows( seq(1:22), predicted.lower, seq(1:22), predicted.upper, code=3, angle=90, length=0.1, col='dark grey')

par(new=T)

predicted.mean<-c( c(y.predicted.north.min[1,1], y.predicted.east.min[1,1], y.predicted.south.min[1,1],y.predicted.west.min[1,1]) , space, c(y.predicted.north.min[2,1], y.predicted.east.min[2,1], y.predicted.south.min[2,1],y.predicted.west.min[2,1]) , space, c(y.predicted.north.min[3,1], y.predicted.east.min[3,1], y.predicted.south.min[3,1],y.predicted.west.min[3,1]) , space, c(y.predicted.north.min[4,1], y.predicted.east.min[4,1], y.predicted.south.min[4,1],y.predicted.west.min[4,1]) )

predicted.SE<-c( c(y.predicted.north.min[1,2], y.predicted.east.min[1,2], y.predicted.south.min[1,2],y.predicted.west.min[1,2]) , space, c(y.predicted.north.min[2,2], y.predicted.east.min[2,2], y.predicted.south.min[2,2],y.predicted.west.min[2,2]) , space, c(y.predicted.north.min[3,2], y.predicted.east.min[3,2], y.predicted.south.min[3,2],y.predicted.west.min[3,2]) , space, c(y.predicted.north.min[4,2], y.predicted.east.min[4,2], y.predicted.south.min[4,2],y.predicted.west.min[4,2]) )

predicted.lower<- predicted.mean -predicted.SE

predicted.upper<- predicted.mean+ predicted.SE

plot(predicted.mean, ylim=c(0,50) , col='dark grey',yaxs="i", xaxt='n',xlab=' Time period', ylab= 'Location temperature (°C)', **axis.lty=1, cex.lab=1.2 , cex.names=1.2, font.lab=1.1, cex.axis=1.2** , las=1)

text(c( c(y.predicted.north.min[1,1], y.predicted.east.min[1,1], y.predicted.south.min[1,1],y.predicted.west.min[1,1]) , space, c(y.predicted.north.min[2,1], y.predicted.east.min[2,1], y.predicted.south.min[2,1],y.predicted.west.min[2,1]) , space, c(y.predicted.north.min[3,1], y.predicted.east.min[3,1], y.predicted.south.min[3,1],y.predicted.west.min[3,1]) , space, c(y.predicted.north.min[4,1], y.predicted.east.min[4,1], y.predicted.south.min[4,1],y.predicted.west.min[4,1]) )+3, labels=orientation)

arrows( seq(1:22), predicted.lower, seq(1:22), predicted.upper, code=3, angle=90, length=0.1, col='dark grey')

axis(1, labels=c('0:00-6:00','6:00-12:00','12:00-18:00', '18:00-24:00'), at=c(2.5,8.5,14.5,20.5), tick=FALSE)

abline(40.5, 0, lty=3) ; abline(36, 0, lty=3) ; abline(0,0)

**# 3) NATURAL NESTS EXP**

data<-read.table(**'natural nests exp.csv**', header=T, sep=';')

attach(data)

Temperature<-as.numeric(as.character(Temperature))

nest.ID<-as.factor(Nest.ID)

day<-as.factor(day)

atm.T_C<- (atm.T- mean(atm.T, na.rm=T))/ (2*sd(atm.T, na.rm=T))

Nest.exp<-recode(Nest.type, " 'experiment'=1; else=0 ", as.numeric.result=T, as.factor.result=F ); Nest.exp_C<-Nest.exp-mean(Nest.exp)

morning<-levels(time.cat)[4]

afternoon<-levels(time.cat)[2]

evening<-levels(time.cat)[3]

night<-levels(time.cat)[1]

morning<-recode(time.cat, "morning=1 ; else=0 ", as.numeric.result=T, as.factor.result=F )

afternoon<-recode(time.cat, "afternoon=1 ; else=0 ", as.numeric.result=T, as.factor.result=F )

evening<-recode(time.cat, "evening=1 ; else=0 ", as.numeric.result=T, as.factor.result=F )

morning_C<-morning-mean(morning)

afternoon_C<-afternoon-mean(afternoon)

evening_C<-evening-mean(evening)

library(nlme)

library(AIccmodavg)

model.1<- gls( Temperature~ 1 + atm.T_C+ I(atm.T_C^2)+I(atm.T_C^2)+ I(atm.T_C^3)+ morning_C+ afternoon_C+evening_C , correlation=corCompSymm(form=~1|nest.ID+ 1|day), weights=varFixed(~atm.T_C) )

AICc(model.1)

model.2<- gls( Temperature~ 1 + atm.T_C+ I(atm.T_C^2)+I(atm.T_C^2)+ I(atm.T_C^3)+ Nest.exp_C+morning_C+ afternoon_C+evening_C , correlation=corCompSymm(form=~1|nest.ID+ 1|day), weights=varFixed(~atm.T_C) )

AICc(model.2)

model.3<- gls( Temperature~ 1 + atm.T_C+ I(atm.T_C^2)+I(atm.T_C^2)+ I(atm.T_C^3)+ morning_C+ afternoon_C+evening_C +Nest.exp_C+ Nest.exp_C:atm.T_C , correlation=corCompSymm(form=~1|nest.ID+ 1|day), weights=varFixed(~atm.T_C) )

AICc(model.3)

model.4<- gls( Temperature~ 1 + atm.T_C+ I(atm.T_C^2)+I(atm.T_C^2)+ I(atm.T_C^3)+ Nest.exp_C+morning_C+ afternoon_C+evening_C **+**morning_C:atm.T_C+ afternoon_C:atm.T_C+evening_C:atm.T_C, correlation=corCompSymm(form=~1|nest.ID+ 1|day), weights=varFixed(~atm.T_C) )

AICc(model.4)

model.5<- gls( Temperature~ 1 + atm.T_C+ I(atm.T_C^2)+I(atm.T_C^2)+ I(atm.T_C^3)+ Nest.exp_C+morning_C+ afternoon_C+evening_C +morning_C:Nest.exp_C+ afternoon_C:Nest.exp_C+evening_C:Nest.exp_C, correlation=corCompSymm(form=~1|nest.ID+ 1|day), weights=varFixed(~atm.T_C) )

AICc(model.5)

model.6<- gls( Temperature~ 1 + atm.T_C+ I(atm.T_C^2)+I(atm.T_C^2)+ I(atm.T_C^3)+ Nest.exp_C+morning_C+ afternoon_C+evening_C +morning_C:Nest.exp_C+ afternoon_C:Nest.exp_C+evening_C:Nest.exp_C+morning_C:atm.T_C+ afternoon_C:atm.T_C+evening_C:atm.T_C, correlation=corCompSymm(form=~1|nest.ID+ 1|day), weights=varFixed(~atm.T_C) )

AICc(model.6)

model.7<- gls( Temperature~ 1 + atm.T_C+ I(atm.T_C^2)+I(atm.T_C^2)+ I(atm.T_C^3)+ Nest.exp_C+morning_C+ afternoon_C+evening_C +morning_C:Nest.exp_C+ afternoon_C:Nest.exp_C+evening_C:Nest.exp_C+morning_C:atm.T_C+ afternoon_C:atm.T_C+evening_C:atm.T_C+morning_C:Nest.exp_C:atm.T_C+ afternoon_C:Nest.exp_C :atm.T_C +evening_C:Nest.exp_C :atm.T_C , correlation=corCompSymm(form=~1|nest.ID+ 1|day), weights=varFixed(~atm.T_C) )

AICc(model.7)

model.8<- gls( Temperature~ 1 + atm.T_C+ I(atm.T_C^2)+I(atm.T_C^2)+ I(atm.T_C^3)+ Nest.exp_C+morning_C+ afternoon_C+evening_C +morning_C:Nest.exp_C+ afternoon_C:Nest.exp_C+evening_C:Nest.exp_C+morning_C:atm.T_C+ afternoon_C:atm.T_C+evening_C:atm.T_C+ Nest.exp_C:atm.T_C, correlation=corCompSymm(form=~1|nest.ID+ 1|day), weights=varFixed(~atm.T_C) )

AICc(model.8)

summary(model.6)

Confint(model.6)

**# Figures**

**# treatment * time day – T=mean x time of the day**

newdata.controls<-data.frame (atm.T_C= c(tapply(atm.T_C, time.cat, mean)[1], tapply(atm.T_C, time.cat, mean)[4], tapply(atm.T_C, time.cat, mean)[2],tapply(atm.T_C, time.cat, mean)[3]), Nest.exp_C= rep( min(Nest.exp_C), 4 ) , morning_C=c(min(morning_C), max(morning_C), min(morning_C), min(morning_C)), afternoon_C=c(min(afternoon_C), min(afternoon_C), max(afternoon_C), min(afternoon_C)), evening_C=c(min(evening_C), min(evening_C), min(evening_C), max(evening_C)) )

y.predicted.controls.mean<- predictSE.gls( mod=model.6, newdata=newdata.controls, se.fit=TRUE, print.matrix=T)

newdata.exp<-data.frame (atm.T_C= c(tapply(atm.T_C, time.cat, mean)[1], tapply(atm.T_C, time.cat, mean)[4], tapply(atm.T_C, time.cat, mean)[2],tapply(atm.T_C, time.cat, mean)[3]), Nest.exp_C= rep( max(Nest.exp_C), 4 ) , morning_C=c(min(morning_C), max(morning_C), min(morning_C), min(morning_C)), afternoon_C=c(min(afternoon_C), min(afternoon_C), max(afternoon_C), min(afternoon_C)), evening_C=c(min(evening_C), min(evening_C), min(evening_C), max(evening_C)) )

y.predicted.exp.mean<- predictSE.gls(mod= model.6, newdata=newdata.exp, se.fit=TRUE, print.matrix=T)

abline(40.5, 0, col='red'); abline(36, 0,col='blue')

space<-c(-10,-10)

predicted.mean<-c( c(y.predicted.controls.mean[1,1], y.predicted.exp.mean[1,1]) , space, c(y.predicted.controls.mean[2,1], y.predicted.exp.mean[2,1]), space, c(y.predicted.controls.mean[3,1], y.predicted.exp.mean[3,1]), space, c(y.predicted.controls.mean[4,1], y.predicted.exp.mean[4,1]))

predicted.SE<-c( c(y.predicted.controls.mean[1,2], y.predicted.exp.mean[1,2]) , space, c(y.predicted.controls.mean[2,2], y.predicted.exp.mean[2,2]), space, c(y.predicted.controls.mean[3,2], y.predicted.exp.mean[3,2]), space, c(y.predicted.controls.mean[4,2], y.predicted.exp.mean[4,2]))

predicted.lower<- predicted.mean -predicted.SE

predicted.upper<- predicted.mean+ predicted.SE

plot(predicted.mean, ylim=c(12,21) , xlab='', xaxt='n', col=c('dark grey', 'black', 'white', 'white', 'dark grey', 'black', 'white', 'white','dark grey', 'black', 'white', 'white','dark grey', 'black'), pch=19, ylab='', **axis.lty=1, cex.lab=1.2 , cex.names=1.2, font.lab=1.1, cex.axis=1.2** , las=1)

arrows( seq(1:14), predicted.lower, seq(1:14), predicted.upper, code=3, angle=90, length=0.05, col=c('dark grey', 'black', 'white', 'white', 'dark grey', 'black', 'white', 'white','dark grey', 'black', 'white', 'white','dark grey', 'black') )

par(new=TRUE)

plot(predicted.mean, ylim=c(12,21) , xaxt='n', col=c('dark grey', 'black', 'white', 'white', 'dark grey', 'black', 'white', 'white','dark grey', 'black', 'white', 'white','dark grey', 'black'), pch=19, xlab=' Time period', ylab= 'Location temperature (°C)', **axis.lty=1, cex.lab=1.2 , cex.names=1.2, font.lab=1.1, cex.axis=1.2** , las=1 )

axis(1, labels=c('0:00-6:00','6:00-12:00','12:00-18:00', '18:00-24:00'), at=c(1.5,5.5,9.5,13.5), tick=FALSE)

**# 4) TEMPERATURE EXP CHAMBERS**

data<-read.table('experimental chambers.csv', header=T, sep=',')

attach(data)

date.ID<-as.factor(day)

natural.nest<-recode(nest.type, " 'nestbox'=0; else=1 ", as.numeric.result=T, as.factor.result=F ); natural.nest_C<-natural.nest-mean(natural.nest)

sun<-recode(treatment, " 'sun'=1; else=0 ", as.numeric.result=T, as.factor.result=F ); sun_C<-sun-mean(sun)

library(lme4)

library(AICcmodavg)

model.chambers.0<- lmer(Temperature~ 1 + (1|date.ID) + sun_C)

AICc(model.chambers.0)

model.chambers.1<- lmer(Temperature~ 1 + (1|date.ID) + natural.nest_C+ sun_C)

AICc(model.chambers.1)

model.chambers.2<- lmer(Temperature~ 1 + (1|date.ID) + natural.nest_C*sun_C)

AICc(model.chambers.2)

summary(model.chambers.1)

**# figures**

library(AICcmodavg)

**# treatment * nest type**

newdata.naturalnests<-data.frame (atm.T_C= rep(0,2), natural.nest_C= rep( max(natural.nest_C), 2 ) , sun_C=c(min(sun_C), max(sun_C)) )

y.predicted.naturalnests.mean<- predictSE.mer(mod=model.chambers.1, newdata=newdata.naturalnests, se.fit=TRUE, type='response', level=0, print.matrix=T)[,1]

y.predicted.naturalnests.SE<- predictSE.mer(mod= model.chambers.1, newdata=newdata.naturalnests, se.fit=TRUE, type='response', level=0, print.matrix=T)[,2]

y.predicted.naturalnests.upper<-y.predicted.naturalnests.mean+y.predicted.naturalnests.SE

y.predicted.naturalnests.lower<-y.predicted.naturalnests.mean-y.predicted.naturalnests.SE

newdata.nestboxes<-data.frame (atm.T_C= rep(0,2), natural.nest_C= rep( min(natural.nest_C), 2 ) , sun_C=c(min(sun_C), max(sun_C)) )

y.predicted.nestboxes.mean<- predictSE.mer( mod=model.chambers.1, newdata=newdata.nestboxes, se.fit=TRUE, type='response', level=0, print.matrix=T)[,1]

y.predicted.nestboxes.SE<- predictSE.mer( mod=model.chambers.1, newdata=newdata.nestboxes, se.fit=TRUE, type='response', level=0, print.matrix=T)[,2]

y.predicted.nestboxes.upper<-y.predicted.nestboxes.mean+y.predicted.nestboxes.SE

y.predicted.nestboxes.lower<-y.predicted.nestboxes.mean-y.predicted.nestboxes.SE

space<-c(-10,-10)

plot(c( c(y.predicted.naturalnests.mean[1], y.predicted.nestboxes.mean[1]) , space, c(y.predicted.naturalnests.mean[2], y.predicted.nestboxes.mean[2]) ), ylim=c(25,36) , xlab=' Chamber type', xaxt='n', pch=c(19,19,3,3,19,19,3,3), ylab='Chamber temperature (°C)' , col=c('dark gray','black', 'white', 'white', 'dark gray', 'black', 'white', 'white' ),**axis.lty=1, cex.lab=1.2 , cex.names=1.2, font.lab=1.1, cex.axis=1.2** , las=1)

arrows( c(1, 5), y.predicted.naturalnests.lower, c(1,5),y.predicted.naturalnests.upper, code=3, angle=90, length=0.05,col='dark gray' )

arrows( c(2, 6), y.predicted.nestboxes.lower, c(2,6),y.predicted.nestboxes.upper, code=3, angle=90, length=0.05,col='black' )

axis(1, labels=c('shade', 'sun'), at=c(1.5, 5.5), tick=FALSE, cex.axis=1.2)

**# 5) NORMAL LETHAL TIME**

library(car)

library(pscl)

data<-read.table('**normal-lethal.csv**', header=T, sep=',')

data<-data[2:35,]

attach(data)

Time.below<- 24-(Time.normal+Time.lethal)

Time.above<-(24-Time.below)

natural.nest<-recode(nest.type, " 'nestbox'=0; else=1 ", as.numeric.result=T, as.factor.result=F ); natural.nest_C<-natural.nest-mean(natural.nest)

sun<-recode(treatment, " 'sun'=1; else=0 ", as.numeric.result=T, as.factor.result=F ); sun_C<-sun-mean(sun)

**# Time normal**

f1<-formula( Time.normal~treatment+nest.type | treatment+nest.type )

f2<-formula( Time.normal~treatment | treatment)

model.normal.1a<-hurdle(formula=f1, dist='poisson', link='logit')

AIC(model.normal.1a)

model.normal.1b<-hurdle(formula=f1, dist='negbin', link='logit')

AIC(model.normal.1b)

**model.normal.2a<-hurdle(formula=f2, dist='poisson', link='logit')**

AIC(model.normal.2a)

model.normal.2b<-hurdle(formula=f2, dist='negbin', link='logit')

AIC(model.normal.2b)

summary(model.normal.2a)

**# Time lethal**

f1<-formula( Time.lethal~treatment + nest.type | treatment + nest.type )

f2<-formula( Time.lethal~treatment | treatment )

model.lethal.1a<-hurdle(formula=f1, dist='poisson', link='logit')

AIC(model.lethal.1a)

model.lethal.1b<-hurdle(formula=f1, dist='negbin', link='logit')

AIC(model.lethal.1b)

**model.lethal.2a<-hurdle(formula=f2, dist='poisson', link='logit')**

AIC(model.lethal.2a)

model.lethal.2b<-hurdle(formula=f2, dist='negbin', link='logit')

AIC(model.lethal.2b)

summary(model.lethal.2a)

**# 6) HATCHING TIME**

data<-read.table('**Incubation length.csv**', header=T, sep=';')

attach(data)

library(car)

library(lme4)

library(AICcmodavg)

natural.nest<-recode(chamber.type, " 'Natural nest'=1; else=0 ", as.numeric.result=T, as.factor.result=F ); natural.nest_C<-natural.nest-mean(natural.nest)

sun<-recode(treatment, " 'Sun'=1; else=0 ", as.numeric.result=T, as.factor.result=F ); sun_C<-sun-mean(sun)

egg.number_C<- (egg.number- mean(egg.number, na.rm=T))/ (2*sd(egg.number, na.rm=T))

Nest.ID<-as.factor(Nest.ID)

model.hatching.0<- lmer(Incubation.length~ 1+ (1|Nest.ID) )

AICc(model.hatching.0)

model.hatching.1<- lmer(Incubation.length~ 1+ (1|Nest.ID) + egg.number_C )

AICc(model.hatching.1)

**model.hatching.2<- lmer(Incubation.length~ 1+ (1|Nest.ID) + egg.number_C + sun_C )**

AICc(model.hatching.2)

model.hatching.3<- lmer(Incubation.length~ 1+ (1|Nest.ID) + egg.number_C +sun_C + natural.nest_C )

AICc(model.hatching.3)

model.hatching.4<- lmer(Incubation.length~ 1+ (1|Nest.ID) + egg.number_C + sun_C*natural.nest_C )

AICc(model.hatching.4)

model.hatching.5<- lmer(Incubation.length~ 1+ (1|Nest.ID) + egg.number_C *sun_C )

AICc(model.hatching.5)

summary(model.hatching.2)

**# Figures**

**#Chamber type**

newdata<-data.frame ( egg.number_C= rep(0, 2 ) , sun_C=c(min(sun_C), max(sun_C)) )

y.predicted.mean<- predictSE.mer( mod=model.hatching.2, newdata=newdata, se.fit=TRUE, type='response', level=0, print.matrix=T)[,1]

y.predicted.SE<- predictSE.mer( mod=model.hatching.2, newdata=newdata, se.fit=TRUE, type='response', level=0, print.matrix=T)[,2]

y.predicted.upper<-y.predicted.mean+y.predicted.SE

y.predicted.lower<-y.predicted.mean-y.predicted.SE

abline(40.5, 0, col='red'); abline(36, 0,col='blue'); space<-c(-10,-10)

barplot(y.predicted.mean, ylim=c(12,14) , xlab='Chamber type', ylab='Development time (days)' , names.arg=c('shade (cool)', 'sun (hot)'), xpd=FALSE, **axis.lty=1, cex.lab=1.2 , cex.names=1.2, font.lab=1.1, cex.axis=1.0** , las=1 )

arrows( c(0.7, 1.9), y.predicted.lower, c(0.7, 1.9),y.predicted.upper, code=3, angle=90, length=0.5)

abline(12,0)

**# egg number**

newdata<-data.frame ( egg.number_C= sort(unique(egg.number_C)) , sun_C=rep(0,5) )

y.predicted.mean<- predictSE.mer( mod=model.hatching.2, newdata=newdata, se.fit=TRUE, type='response', level=0, print.matrix=T)[,1]

y.predicted.SE<- predictSE.mer( mod=model.hatching.2 newdata=newdata, se.fit=TRUE, type='response', level=0, print.matrix=T)[,2]

y.predicted.upper<-y.predicted.mean+y.predicted.SE

y.predicted.lower<-y.predicted.mean-y.predicted.SE

abline(40.5, 0, col='red'); abline(36, 0,col='blue'); space<-c(-10,-10)

barplot(y.predicted.mean, ylim=c(12,14) , xlab='Egg number', ylab='Development time (days)' , xpd=FALSE, **axis.lty=1, cex.lab=1.2 , cex.names=1.2, font.lab=1.1, cex.axis=1.0** , las=1 )

arrows( c(0.7, 1.9, 3.1, 4.3,5.5), y.predicted.lower, c(0.7, 1.9, 3.1, 4.3,5.5),y.predicted.upper, code=3, angle=90, length=0.2)

abline(12,0)

***# Daily Temperatures FG***

**data<-read.table('temperature FG.csv', header=T, sep=';')**

**# Mean x day**

Tmax.day_mean<-tapply(T.max, day.year, mean, na.rm=T)

Tmax.day_sd<-tapply(T.max, day.year, sd, na.rm=T)

Tmax.day_n<-tapply(T.max, day.year, length)

Tmax.day_se<-Tmax.day_sd/sqrt(Tmax.day_n)

Tmax.day_upper<-Tmax.day_mean+Tmax.day_se

Tmax.day_lower<-Tmax.day_mean-Tmax.day_se

Tmin.day_mean<-tapply(T.min, day.year, mean, na.rm=T)

Tmin.day_sd<-tapply(T.min, day.year, sd, na.rm=T)

Tmin.day_n<-tapply(T.min, day.year, length)

Tmin.day_se<-Tmin.day_sd/sqrt(Tmin.day_n)

Tmin.day_upper<-Tmin.day_mean+Tmin.day_se

Tmin.day_lower<-Tmin.day_mean-Tmin.day_se

**# PLOT**

plot(Tmax.day_mean, cex=0.1, xlim=c(1,366), ylim=c(min(Tmin.day_lower), max(Tmax.day_upper) ), xlab='', ylab='')

lines(Tmax.day_mean, xlim=c(1,366))

lines(Tmax.day_upper, lty=2, xlim=c(1,366))

lines(Tmax.day_lower, lty=2, xlim=c(1,366))

par(new=T)

plot(Tmin.day_mean, cex=0.1, xlim=c(1,366), ylim=c(min(Tmin.day_lower), max(Tmax.day_upper) ), xlab= 'day (0-365)', ylab='temperature')

lines(Tmin.day_mean, xlim=c(1,366))

lines(Tmin.day_upper, lty=2, xlim=c(1,366))

lines(Tmin.day_lower, lty=2, xlim=c(1,366))

**#Mean x week**

Tmax.day_mean<-tapply(T.max, week, mean, na.rm=T)

Tmax.day_sd<-tapply(T.max, week, sd, na.rm=T)

Tmax.day_n<-tapply(T.max, week, length)

Tmax.day_se<-Tmax.day_sd/sqrt(Tmax.day_n)

Tmax.day_upper<-Tmax.day_mean+Tmax.day_se

Tmax.day_lower<-Tmax.day_mean-Tmax.day_se

Tmin.day_mean<-tapply(T.min, week, mean, na.rm=T)

Tmin.day_sd<-tapply(T.min, week, sd, na.rm=T)

Tmin.day_n<-tapply(T.min, week, length)

Tmin.day_se<-Tmin.day_sd/sqrt(Tmin.day_n)

Tmin.day_upper<-Tmin.day_mean+Tmin.day_se

Tmin.day_lower<-Tmin.day_mean-Tmin.day_se

**# PLOT**

plot(Tmax.day_mean, cex=0.1, xlim=c(1,max(week)), ylim=c(min(Tmin.day_lower), max(Tmax.day_upper) ), xlab= '', ylab='Temperature', xaxt='n')

axis( side=1, labels=c('January', 'February', 'March', 'April', 'May', 'June', 'July', 'August', 'September', 'October', 'November', 'December') , at=seq(1,max(week),4.7) )

lines(Tmax.day_mean, xlim=c(1,max(week)))

lines(Tmax.day_upper, lty=2, xlim=c(1,max(week)))

lines(Tmax.day_lower, lty=2, xlim=c(1,max(week)))

par(new=T)

plot(Tmin.day_mean, cex=0.1, xlim=c(1,max(week)), ylim=c(min(Tmin.day_lower), max(Tmax.day_upper) ), xlab= '', ylab='Temperature', xaxt='n')

lines(Tmin.day_mean, xlim=c(1,max(week)))

lines(Tmin.day_upper, lty=2, xlim=c(1,max(week)))

lines(Tmin.day_lower, lty=2, xlim=c(1,max(week)))

**#Mean x week x year**

Tmax.day_mean<-tapply(T.max, week.year, mean, na.rm=T)

Tmax.day_sd<-tapply(T.max, week.year, sd, na.rm=T)

Tmax.day_n<-tapply(T.max, week.year, length)

Tmax.day_se<-Tmax.day_sd/sqrt(Tmax.day_n)

Tmax.day_upper<-Tmax.day_mean+Tmax.day_se

Tmax.day_lower<-Tmax.day_mean-Tmax.day_se

Tmin.day_mean<-tapply(T.min, week.year, mean, na.rm=T)

Tmin.day_sd<-tapply(T.min, week.year, sd, na.rm=T)

Tmin.day_n<-tapply(T.min, week.year, length)

Tmin.day_se<-Tmin.day_sd/sqrt(Tmin.day_n)

Tmin.day_upper<-Tmin.day_mean+Tmin.day_se

Tmin.day_lower<-Tmin.day_mean-Tmin.day_se

**# PLOT**

plot(Tmax.day_mean, cex=0.1, xlim=c(1,max(week.year)), ylim=c(min(Tmin.day_lower,na.rm=T), max(Tmax.day_upper,na.rm=T) ), xlab= '', ylab='Temperature', xaxt='n')

axis( side=1, labels=c('2005', '2006', '2007', '2008', '2009', '2010', '2011', '2012', '2013') , at=(seq(1,max(week.year),12*4.8) ) )

lines(Tmax.day_mean, xlim=c(1,max(week)))

lines(Tmax.day_upper, lty=2, xlim=c(1,max(week)))

lines(Tmax.day_lower, lty=2, xlim=c(1,max(week)))

par(new=T)

plot(Tmin.day_mean, cex=0.1, xlim=c(1,max(week.year)), ylim=c(min(Tmin.day_lower,na.rm=T), max(Tmax.day_upper,na.rm=T) ), xlab= '', ylab='Temperature', xaxt='n')

lines(Tmin.day_mean, xlim=c(1,max(week)))

lines(Tmin.day_upper, lty=2, xlim=c(1,max(week)))

lines(Tmin.day_lower, lty=2, xlim=c(1,max(week)))
