# Supplementary material for: High atmospheric temperatures and ‘ambient incubation’ drive embryonic development and lead to earlier hatching in a passerine bird
Source: R Soc Open Sci. 2016 Feb 3;3(2):150371. doi: 10.1098/rsos.150371 (PMC4785966; doi:10.1098/rsos.150371)
Supplement: additional tables for the GLMM analyses [file rsos150371supp2.docx]

**Table S1**. Candidate models for location temperature (‘T’) across location types and time of day, controlled for atmospheric temperature (‘atm.T’).

| **Model** | **AICc** | **ΔAICc** |
| --- | --- | --- |
| **T ~ 1 + atm.T + atm.T^2^ + atm.T^3^ + time of day + location + time of day:location** | **142652** | **0** |
| T ~ 1 + atm.T + atm.T^2^ + atm.T^3^ + time of day + location + time of day:location+ location:atm.T | 142657 | 5 |
| T ~ 1 + atm.T + atm.T^2^ + atm.T^3^ + time of day + location + location:atm.T | 143291 | 639 |
| T ~ 1 + atm.T + atm.T^2^ + atm.T^3^ + location + location:atm.T | 156655 | 14003 |

The superscripts ‘^2^’ and ‘^3^’ indicate quadratic and cubic effects respectively. ‘:’ symbolize interactions; ‘AICc’ represents the Akaike information criterion corrected for sample size; ΔAICc is the difference in AICc scores between focal models and the best candidate model (model with the lowest AICc score; in **bold** characters)

**Table S2. a)** Candidate models for temperature of natural nests (‘T’) according to nest orientation, time of day, and nest height, controlled for atmospheric temperature (‘atm.T’). **b)** Candidate models for temperature of natural nests according to nest structure (treatment: ‘roof removed’ vs ‘control’) and time of day, controlled for atmospheric temperature (‘atm.T’).

**a)**

| **Model** | **AICc** | **ΔAICc** |
| --- | --- | --- |
| **T ~ 1 + atm.T + atm.T^2^ + atm.T^3^ + time of day + orientation + orientation:time of day + atm.T:time of day** | **16607** | **0** |
| T ~ 1 + atm.T + atm.T^2^ + atm.T^3^ + time of day + orientation + orientation:time of day + atm.T:time of day + height + height:time of day | 16608 | 1 |
| T ~ 1 + atm.T + atm.T^2^ + atm.T^3^ + time of day + orientation + orientation:time of day + atm.T:time of day + height | 16612 | 5 |
| T ~ 1 + atm.T + atm.T^2^ + atm.T^3^ + t + atm.T:time of day + orientation + orientation:time of day + height + height:time of day | 16643 | 36 |
| T ~ 1 + atm.T + atm.T^2^ + atm.T^3^ + time of day + orientation + orientation:time of day + height + height:time of day + atm.T:orientation | 16643 | 36 |
| T ~ 1 + atm.T + atm.T^2^ + atm.T^3^ + time of day | 16645 | 38 |
| T ~ 1 + atm.T + atm.T^2^ + atm.T^3^ + time of day + atm.T:time of day + orientation | 16824 | 217 |
| T ~ 1 + atm.T + atm.T^2^ + atm.T^3^ + time of day + atm.T:time of day | 16871 | 264 |
| T ~ 1 + atm.T + atm.T^2^ + atm.T^3^ + time of day + orientation + orientation:time of day | 16904 | 297 |

**b)**

| **Model** | **AICc** | **ΔAICc** |
| --- | --- | --- |
| **T ~ 1 + atm.T + atm.T^2^ + atm.T^3^ + time of day + treatment + treatment:time of day + atm.T:time of day** | **5698** | **0** |
| T ~ 1 + atm.T + atm.T^2^ + atm.T^3^ + time of day + treatment + treatment:time of day+ atm.T:time of day + treatment:time of day:atm.T | 5699 | 1 |
| T ~ 1 + atm.T + atm.T^2^ + atm.T^3^ + time of day + treatment + treatment:time of day + atm.T:time of day + treatment:atm.T | 5699 | 1 |
| T ~ 1 + atm.T + atm.T^2^ + atm.T^3^ + time of day + treatment + treatment:time of day | 5722 | 23 |
| T ~ 1 + atm.T + atm.T^2^ + atm.T^3^ + time of day + treatment + atm.T:time of day | 5745 | 47 |
| T ~ 1 + atm.T + atm.T^2^ + atm.T^3^ + time of day + treatment | 5770 | 72 |
| T ~ 1 + atm.T + atm.T^2^ + atm.T^3^ + time of day + treatment + atm.T:treatment | 5770 | 72 |
| T ~ 1 + atm.T + atm.T^2^ + atm.T^3^ + time of day | 5789 | 91 |

The superscripts ‘^2^’ and ‘^3^’ indicate quadratic and cubic effects respectively. ‘:’ symbolize interactions; ‘AICc’ represents the Akaike information criterion corrected for sample size; ΔAICc is the difference in AICc scores between focal models and the best candidate model (model with the lowest AICc score; in **bold** characters)

**Table S3. a)** Candidate models for temperature of nest chambers (‘T’) according to experimental treatment (‘sun’, ‘shade’) and chamber type (‘nest’, ‘nestbox’). **b)** Candidate models for the number of hours in the day during which experimental chambers (‘hot’ and ‘cool’) were at normal incubation temperatures (36-40°C) according to treatment and chamber type. **c)** Candidate models for the number of hours in the day during which experimental chambers (‘hot’ and ‘cool’) were at lethal incubation temperatures (above 45°C) according to treatment and chamber type. **d)** Candidate models for hatching time according to experimental treatment, chamber type, and laying order (‘egg number’)

**a)**

| **Model** | **AICc** | **ΔAICc** |
| --- | --- | --- |
| **T~ 1 + (1\|date) + treatment + chamber type** | **4348** | **0** |
| T~ 1 + (1\|date) + treatment + chamber type | 4349 | 1 |
| T~ 1 + (1\|date) + treatment | 4355 | 7 |

**b)**

| **Model** | **AICc** | **ΔAICc** |
| --- | --- | --- |
| **Time normal~ treatment \| treatment** | **161** | **0** |
| Time normal~ treatment + chamber type \| treatment + chamber type | 165 | 4 |

**c)**

| **Model** | **AICc** | **ΔAICc** |
| --- | --- | --- |
| **Time lethal~ treatment \| treatment** | **85** | **0** |
| Time lethal~ treatment + chamber type \| treatment + chamber type | 88 | 3 |

**d)**

| **Model** | **AICc** | **ΔAICc** |
| --- | --- | --- |
| **Hatching time ~ 1 + 1\|nest.ID + egg number+ treatment** | **37** | **0** |
| Hatching time ~ 1 + 1\|nest.ID + egg number+ treatment + egg number:treatment | 41 | 4 |
| Hatching time ~ 1 + 1\|nest.ID + egg number+ treatment + chamber type | 42 | 5 |
| Hatching time ~ 1 + 1\|nest.ID + egg number | 45 | 8 |
| Hatching time ~ 1 + 1\|nest.ID + egg number+ treatment + chamber type + treatment:chamber type | 45 | 8 |
| Hatching time ~ 1 + 1\|nest.ID | 52 | 15 |

The superscripts ‘^2^’ and ‘^3^’ indicate quadratic and cubic effects respectively. ‘:’ symbolize interactions; “|” specifies random intercepts in table a), d); while for hurdle models ( b, c) it separates terms fitted in the truncated Poisson component (left hand side) from terms fitted in the binomial component of the model (right hand side). ‘AICc’ represents the Akaike information criterion corrected for sample size; ΔAICc is the difference in AICc scores between focal models and the best candidate model (model with the lowest AICc score; in **bold** characters)
